# A CRISPR-edited isoform of the AMPK kinase LKB1 improves the response to cisplatin in A549 lung cancer cells

**DOI:** 10.1016/j.jbc.2025.108308

**Published:** 2025-02-13

**Authors:** Matheus Brandemarte Severino, Ana Paula Morelli, Isadora Carolina Betim Pavan, Mariana Camargo Silva Mancini, Mariana Marcela Góis, Rafael Junqueira Borges, Renata Rosseto Braga, Luiz Guilherme Salvino da Silva, Nathalia Quintero-Ruiz, Maíra Maftoum Costa, Wesley de Lima Oliveira, Rosângela Maria Neves Bezerra, Eduardo Rochete Ropelle, Fernando Moreira Simabuco

**Affiliations:** 1 Multidisciplinary Laboratory of Food and Health, School of Applied Sciences, University of Campinas, Limeira, Brazil; 2 Department of Biochemistry, Institute of Chemistry, University of São Paulo, São Paulo, Brazil; 3 Department of Physics and Biophysics, Biosciences Institute, State University of São Paulo, Botucatu, Brazil; 4 Center for Molecular Biology and Genetic Engineering (CBMEG), University of Campinas, Campinas, Brazil; 5 Center for Medicinal Chemistry (CQMED), University of Campinas, Campinas, Brazil; 6 Laboratory of Molecular Biology of Exercise (LaBMEx), School of Applied Sciences, University of Campinas, Limeira, Brazil; 7 Applied Molecular Signaling Laboratory (LabSIMA), Department of Biochemistry, Federal University of São Paulo, São Paulo, Brazil

**Keywords:** autophagy, CRISPR/Cas9, cisplatin, metabolism, *STK11*(LKB1), non-small cell lung cancer (NSCLC)

## Abstract

Lung cancer presents the highest mortality rate in the world when compared to other cancer types and often presents chemotherapy resistance to cisplatin. The A549 nonsmall cell lung cancer line is widely used as a model for lung adenocarcinoma studies since it presents a high proliferative rate and a nonsense mutation in the *STK11* gene. The LKB1 protein, encoded by the *STK11* gene, is one of the major regulators of cellular metabolism through AMPK activation under nutrient deprivation. Mutation in the *STK11* gene in A549 cells potentiates cancer hallmarks, such as deregulation of cellular metabolism, aside from the Warburg effect, mTOR activation, autophagy inhibition, and NRF2 and redox activation. In this study, we investigated the integration of these pathways associated with the metabolism regulation by LKB1/AMPK to improve cisplatin response in the A549 cell line. We first used the CRISPR/Cas9 system to generate cell lines with a CRISPR-edited LKB1 isoform (called Super LKB1), achieved through the introduction of a +1 adenine insertion in the first exon of the *STK11* gene after NHEJ-mediated repair. This insertion led to the expression of a higher molecular weight protein containing an alternative exon described in the Peutz-Jeghers Syndrome. Through metabolic regulation by Super LKB1 expression and AMPK activation, we found an increase in autophagy flux (LC3 GFP/RFP *p* < 0.05), as well as a reduction in the phosphorylation of mTORC1 downstream targets (S6K2 phospho-serine 423; *p* < 0.05; and S6 ribosomal protein phospho-serine 240/244; *p* < 0.03). The NRF2 protein exhibited increased levels and more nuclear localization in A549 WT cells compared to the edited cells (*p* < 0.01). We also observed lower levels of H_2_O_2_ in the WT A549 cells, as a possible result of NRF2 activation, and a higher requirement of cisplatin to achieve the IC_50_ (WT: 10 μM; c2SL+: 5.5 μM; c3SL+: 6 μM). The data presented here suggests that the regulation of molecular pathways by the novel Super LKB1 in A549 cells related to metabolism, mTORC1, and autophagy promotes a better response of lung cancer cells to cisplatin. This NHEJ-CRISPR–based approach may be potentially used for lung cancer gene therapy.

Lung cancer has the highest cancer mortality rate (1) and represents 85% of cancer cases in men and women, with lung adenocarcinoma being the most frequent type (2). From lung cancer diagnosis, the standard treatment is chemotherapy with cisplatin. However, non-small cell lung cancer (NSCLC) often presents resistance to chemotherapy and causes most lung cancer patients to progress to death (3).

Lung cancer adenocarcinomas are predominantly associated with the following: Kirsten rat sarcoma viral oncogene homolog (*KRAS* - 32%); epidermal grow factor receptor (11%), and V-Raf murine sarcoma viral oncogene homolog B (*BRAF* - 7%) driver mutations. Additionally, molecular profiling of lung adenocarcinoma has identified a high frequency of mutations in the tumor suppressors serine threonine kinase 11 (*STK11*–17%) and Kelch-like ECH associated protein 1 (*KEAP1* - 17%), based on the analysis of 230 samples from the TCGA database (4). A cohort study has demonstrated that mutations in tumor suppressors, such as *STK11*, are correlated with a poor prognosis for malignant tumors. These mutations can also contribute to increased resistance to platinum-based chemotherapy and reduced efficacy of immunotherapies which include immune checkpoint blockade, such as anti-PDL1 treatment (5).

The *STK11* gene encodes the liver kinase B1 (LKB1) protein. This gene spans more than 20 kb and is composed of 10 exons, nine of them comprising 433 amino acids of a translated protein. The LKB1 is a heterotrimeric complex that is allosterically activated by the STE20-related kinase adapter protein alpha (STRAD) and the adaptor protein calcium-binding protein 39 (CAB39/MO25). LKB1 is an important tumor suppressor and an upstream activator of AMP-activated protein kinase (AMPK) (6).

The LKB1–AMPK axis is activated by an increase in intracellular AMP and ATP depletion (7). AMPK is a well-studied protein that plays a central role in cellular metabolism, regulating several molecular pathways, among them: glycolytic and oxidative metabolism, mammalian target of Rapamycin (mTOR), cell cycle, autophagy, and apoptosis (8). The tumoral suppressor activity of LKB1/AMPK involves a signaling cascade that influences important pathways, and the interplays between these pathways are related to the hallmarks of cancer (9).

*STK11* gene mutations are associated with Peutz-Jeghers syndrome (PJS). Approximately 70% of individuals with the syndrome carry mutations in the *STK11* gene, leading to a loss of LKB1 function (10, 11). This loss can promote the development of polyps, which have the potential to become cancerous. In lung adenocarcinoma, the prevalence of mutations in the *STK11* gene can reach 30% (12, 13). A549 lung cancer cells harbor a mutation in the first exon of the *STK11* gene (c.109C > T), resulting in a premature termination codon (PTC), effectively making this cell line knockout for the canonical LKB1 (12).

On the other hand, a nontranslated LKB1 transcript, containing the insertion of 131 nucleotides as a new exon between exons 1 and 2, was described in PJS patients (14). Recent studies reported that the A549 cells present this transcript, and this alternative exon could function as an alternative start codon for a novel mitochondria-localized LKB1 variant (15). This new variant presents different functions compared with the canonical LKB1. While the mitochondria-localized LKB1 seems to regulate the redox balance in A549 cells, the canonical LKB1 through the AMPK activation regulates pathways related to metabolism and tumor suppression (16).

Using the CRISPR/Cas9 system, we targeted *STK11* to generate cell lines with mutated LKB1, obtaining functional mRNA like the canonical isoform. Tuladhar et al. recently demonstrated that CRISPR/Cas9-induced DNA breaks, repaired by nonhomologous end joining (NHEJ), can lead to INDELs producing mutated mRNAs and proteins. Specifically, after the *STK11* gene editing by CRISPR, they generated a higher molecular weight isoform of LKB1 named Super LKB1, due to the +1/−2 INDELs, and the presence of an alternative exon (+131 bp). The authors discuss that this event could be probably triggered by the regulation of alternative splicing by editing an exon splicing enhancer region. However, the functions of this isoform remained unclear (17).

We, therefore, aimed to edit the previous region of nonsense mutation (Q37∗, also known as c.109C > T) in A549 cells, present in the canonical exon 1 (1a). The insertion of one nucleotide in the edited locus generated a reframed transcript with the alternative exon (1b), thus generating a higher molecular weight LKB1 isoform, similar to the approach employed by Tuladhar et al. Comparing the A549 WT cells to the A549 LKB1-edited cells, we explored some of the molecular pathways associated with LKB1/AMPK signaling and highlighted regulations in mammalian target of rapamycin complex 1 (mTORC1) signaling, autophagy, and redox control.

We found that the A549 WT cells present low activation of AMPK given the lack of LKB1, so we observed a higher activation both in the mTORC1 downstream kinases (S6K1 and S6K2) and the ribosomal S6 protein (S6). We also observed autophagy dysfunction through sequestosome-1 (SQSTM1/p62) accumulation and low autophagy flux. This cellular signaling profile is compatible with noncanonical activation of nuclear erythroid 2-related factor 2 (NRF2). The accumulated p62 (due to mTORC1 phosphorylation) could interact with KEAP1 by KIR domain, sequestrating this protein in positive p62 autophagosomes, which is unable to fuse with lysosomes, thus leading to robust activation of NRF2 (18).

Hence, the regulation of metabolism through LKB1/AMPK may greatly contribute to an increase in autophagy flux and the inhibition of NRF2. Under these conditions, the A549 LKB1-edited cells exhibited an enhanced response to cisplatin. We used metformin as a pharmacological stimulus of autophagy promoted by AMPK activation (19) and 3-methyladenine (3-MA) as an autophagy inhibitor (19). This allowed us to assess the impact of autophagy in conjunction with cisplatin treatment. Consequently, we characterized the Super LKB1 isoform generated through CRISPR/Cas9, elucidating its influence on cellular metabolism and noncanonical NRF2 activation in A549 cells. Our findings highlight the Super LKB1, generated through NHEJ repair mediated by CRISPR/Cas9, as a potential candidate for the development of new cancer treatments. Also, our findings underscore the potential of new anticancer variants of LKB1, opening new avenues for innovative treatments using CRISPR technology to benefit cancer patients or individuals with diseases associated with LKB1 mutations like PJS.

## Results

### The *STK11* gene editing in the A549 cells by CRISPR/Cas9 generated a higher molecular weight LKB1 with an alternative exon

First, we compared the expression of *STK11* variants in healthy lungs (Fig. S1*A*) *versus* lung cancer, which is represented by the A549 cells (Fig. S1*B*), indicating the Q37∗ c.109C>T mutation in lung cancer and the presence of a mitochondrial LKB1 isoform generated by alternative splicing, as previously described (15). The length of the transcripts (Fig. S1*C*) and an edition scheme we designed for the first exon of *STK11* in A549 (Fig. S1*D*) were also shown. Lastly, we present the scheme of the Super LKB1 generated in our experiments by a CRISPR INDEL and the alternative exon incorporation (Fig. S1*E*).

To evaluate the efficiency of the edition by CRISPR/Cas9, the PCR product of the *STK11* genomic locus of the edition was digested with T7E1, demonstrating that the sgRNA/Cas9 group was positive for INDELs (Fig. S2*A*). After clone selection, one WT clone was named cWT, and two clones with LKB1 expression were named clone Super LKB1: c2SL+ and c3SL+, since these clones presented a higher molecular weight band than a positive control (HEK293T), (Fig. S2*B*). The locus of the edition was sequenced from genomic DNA and aligned against cWT (Fig. S2*C*). Both clones c2SL+ and c3SL + presented the same adenine (A) insertion (Fig. S2, *D* and *E*). To evaluate the zygosity of the edition, the chromatograms were decomposed, confirming one single sequence in the chromatogram decomposition and indicating that super LKB1 clones are homozygous for the adenine insertion in both edited clones (Fig. S2, *G* and *H*). The off-targets were evaluated in edited cells in high-prediction regions, and no off-targets were detected in the edited cells' DNA (Fig. S8, *A* and *B*).

To evaluate if the higher molecular weight band of LKB1 detected in Western blotting (Fig. S2*B*) of the c2SL+ and c3SL + clones presented the alternative exon (131 nts) described in the literature (12, 14), RNA extraction was performed followed by complementary DNA (cDNA) synthesis and PCR. A higher molecular weight transcript can be observed specifically in the edited clones after PCR using primers for the 5′UTR region to the exon 4 (Fig. S3*A*). The LKB1 band was cloned into a pGEM-T easy vector (Promega) and sequenced. The sequence showed the presence of the INDEL, a +1 adenine insertion, followed by the A549 mutation (Q37∗; 109C>T) highlighted in red and the alternative exon between exons 1 and 2 (Fig. S3*B*).

After confirming the sequence of the edited transcript, the full amino acids sequence of the Super LKB1 was designed and submitted in the Clustal Omega. The Super LKB1 exhibited a comprehensive translation amino acid sequence devoid of PTCs. In our alignment of amino acid sequences between WT LKB1 and Super LKB1 (Fig. S4*A*), a resemblance in the N-terminal regions of the proteins was observed. Notably, downstream to the INDEL regions, a distinct sequence emerged in Super LKB1 compared to WT, featuring an additional 44 amino acids. Subsequently, from exon 2 to the C-terminal region, the amino acid sequences of the isoforms remained identical, which means that the Super LKB1 can generate a full protein in the A549-edited cells.

We also modeled the canonical LKB1 (Fig. S4*B*) and Super LKB1 (Fig. S4*C*) isoforms with AlphaFold software, an artificial intelligence to predict biological structures. To evaluate the differences between the isoforms, the amino acid structures were then aligned in PyMOL software. The Super LKB1 presents an additional loop (highlighted in the image), probably due to the new sequence in the Super LKB1, in comparison with canonical LKB1 (Fig. S4*D*).

Next, we employed TargetP 2.0 software to predict the localization of the isoforms, focusing on the potential mitochondrial transient peptide. Both the canonical and Super LKB1 isoforms exhibited a higher probability of localization outside the mitochondria, unlike the recently documented mitochondrial LKB1 by Tan *et al.*, 2023. Interestingly, the mitochondrial LKB1 displayed a score ten times higher than mitochondrial localization (Fig. S4*E*). Additionally, MULocDeep software concurred in predicting a similar localization for the canonical and Super LKB1 isoforms (Nucleus/Cytoplasm), which aligns with the cytoplasmic localization of the mitochondrial LKB1 (Fig. S4*F*).

### LKB1 overexpression activates AMPK phosphorylation at 172 threonine residue

To demonstrate the activation of AMPK by LKB1 (20), a canonical LKB1 vector was constructed and named pFLAG-LKB1 for LKB1 overexpression (Fig. S5, *A* and *B*). The A549 WT cells transfected with the pFLAG-LKB1 presented increased AMPK phosphorylation in 172 threonine compared to the FLAG transfection group (FLAG vs FLAG-LKB1∗∗; ∗∗*p* < 0.01) after 72 h.

**Figure 1:**
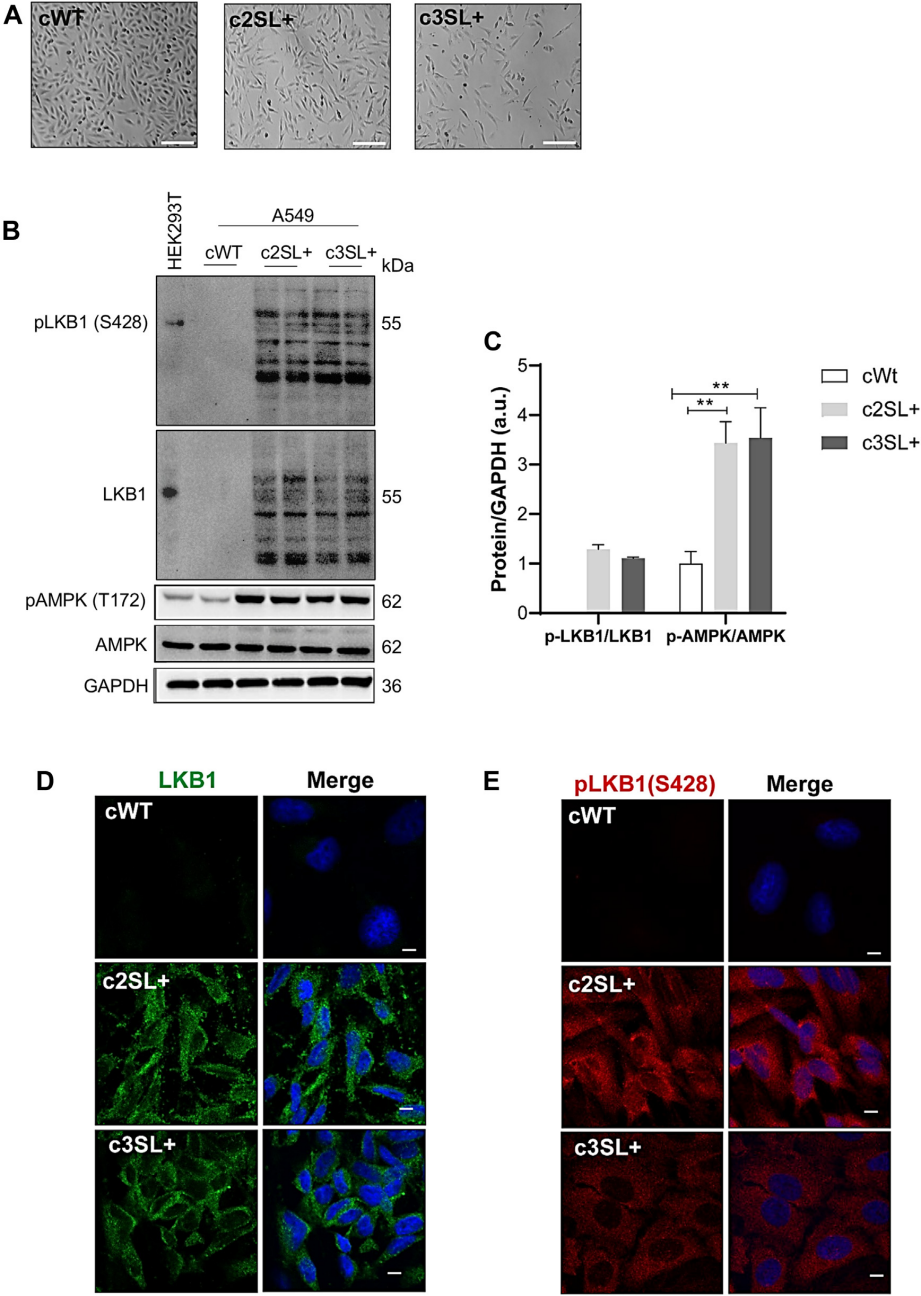
**Super LKB1 enhanced the AMPK activation through the 172-threonine phosphorylation.***A*, optical microscopy photos of cell lines; (*B*) Western blotting of pLKB1 (S428), LKB1, pAMPK (T172), AMPK, and GAPDH in HEK293T used as an LKB1 positive control, cWT, c2SL+, and c3SL + cells; (*C*) normalized levels of proteins by GAPDH (a.u); (*D*) immunofluorescence of LKB1 in cWT, c2SL+, and c3SL + cells; (*E*) immunofluorescence of pLKB1 (S428) in cWT, c2SL+, and c3SL + cells. The data are presented as mean ± SD. Statistical analysis was performed by ANOVA followed by Dunnet’s test ∗*p* < 0.05, ∗∗*p* < 0.01. These data are representative of two independent experiments.

After examining the edited cell lines, we initially conducted optical microscopy imaging of the cells 24 h postseeding (Fig. 1*A*). We subsequently performed protein expression analysis on all edited cells to investigate whether Super LKB1 could enhance AMPK activation through phosphorylation at threonine 172. The edited clones presented higher molecular weight protein bands corresponding to LKB1 and phospho-LKB1 at Serine 428. All edited clones exhibited increased AMPK phosphorylation at the threonine 172 residue (c2SL + vs cWT∗∗, c3SL + vs cWT∗∗) (Fig. 1, *B* and *C*; ∗∗*p* < 0.01). We also assessed the localization of LKB1 and phospho-LKB1 (S428) through confocal fluorescence microscopy. In the edited cells, both LKB1 and pLKB1 were mostly localized in the cytoplasm, with some nuclear signal detected in phospho-LKB1. Conversely, WT cells displayed no antibody staining of LKB1 (Fig. 1, *D* and *E*).

To confirm the interaction of the Super LKB1, the protein–protein interaction network was analyzed by STRING (Fig. S6*A*). As described, LKB1 presents interactions with four AMPK subunits, AMPK is a heterotrimer complex composed of an α catalytic subunit, a β subunit, and a γ regulatory subunit. In the interaction network, the α2 (*PRKAA2*) subunit is highlighted since the 172 threonine residue in the C-lobe kinase domain is phosphorylated by LKB1 (Fig. S6*B*) in human analysis.

To elucidate the potential interactions between the LKB1 isoforms and the AMPK α2 subunit, we initially identified and extracted low-confidence regions in the canonical LKB1, Super LKB1, and AMPK α2 subunit proteins through b-factor coloring. The highly confident regions were subjected to protein-protein docking using ClusPro. Among the top 10 structures generated by ClusPro, we aligned the portion of the AMPK α2 subunit docking with the AMPK complex (PDB: 4CFE). This alignment facilitated the identification of dockings that did not overlap with the regulatory subunits of the AMPK complex, as such overlap might pose a physical impediment to interaction. The selected protein-protein dockings were visualized in surface view using PyMOL for both canonical LKB1 (Fig. S6*C*) and the Super LKB1 (Fig. S6*D*) to enhance the clarity of the interaction. Fig. S6*E* presents the docking of canonical LKB1 aligned with the AMPK complex, and AMPK α2 subunit, highlighting the 172 threonine residue. Similarly, the docking of Super LKB1 aligned with the AMPK complex was illustrated in Fig. S6*F*, along with the interaction of Super LKB1 with the AMPK α2 subunit, emphasizing the 172 threonine residue. The interactions between the LKB1 isoforms and the AMPK α2 subunit were found to be similar.

### Super LKB1 affects the EMT, survival, and migration of A549 cells

**Figure 2:**
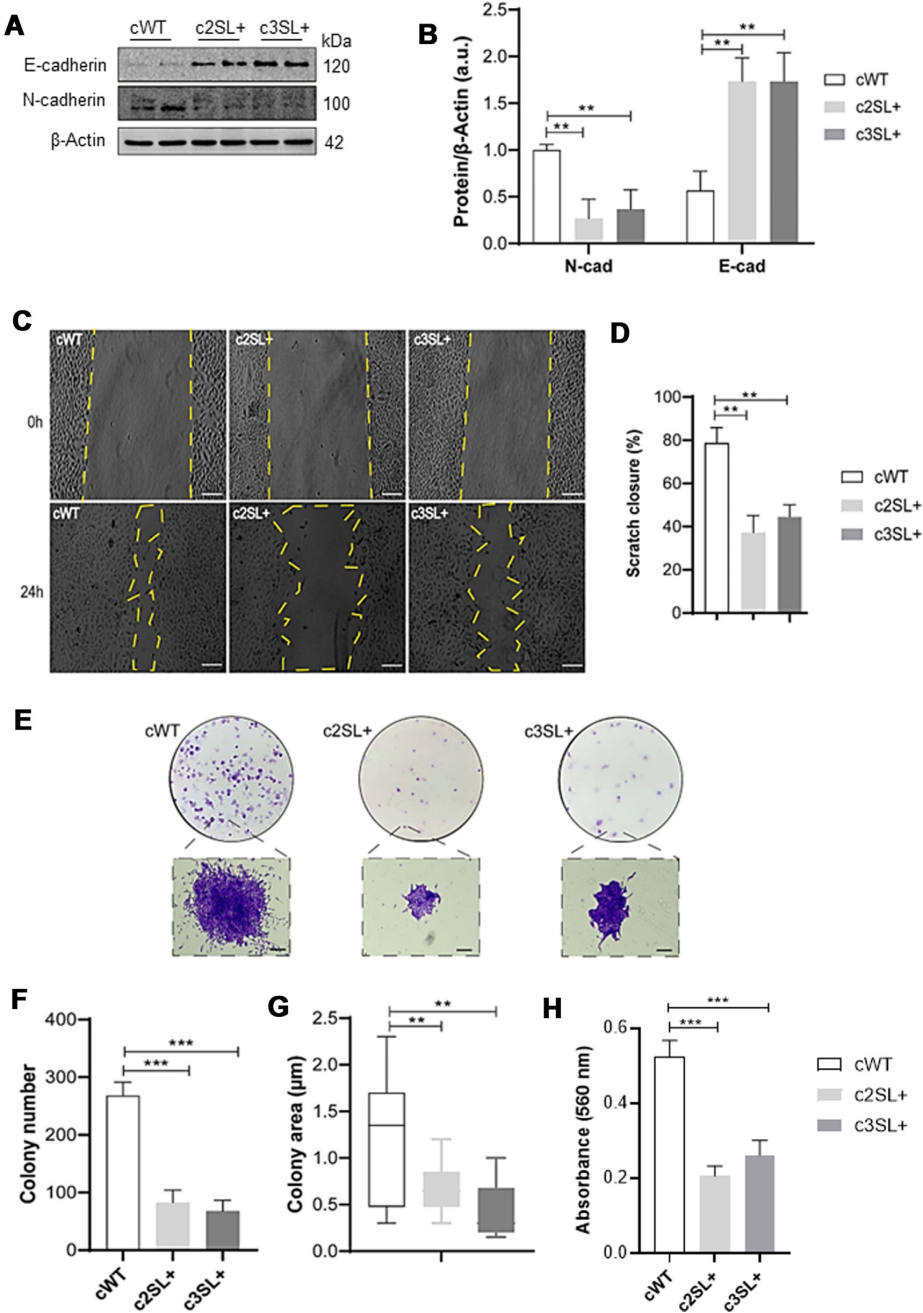
**Super LKB1 affects the epithelium mesenchymal transition, survival, and migration in A549-edited cells.***A*, Western blotting of E-cadherin, N-cadherin, and β-Actin in cWT, c2SL+, and c3SL + cells; (*B*) normalized levels of proteins by β-Actin (a.u); (*C*) scratch assay of cWT, c2SL+, and c3SL + cells treated with 60 μM of mitomycin C for 2 h; (*D*) quantification of scratch area after 24 h of scratch in percentage; (*E*) colony formation of cWT, c2SL+, and c3SL+; (*F*) quantification of colony number in cWT, c2SL+, and c3SL+; (*G*) colony area (μm) in cWT, c2SL+, and c3SL+; (*H*) total absorbance of violet crystal of cWT, c2SL+, and c3SL + cells colonies. Data are presented as mean ± SD. Statistical analysis was performed by ANOVA followed by Dunnet’s test ∗*p* < 0.05, ∗∗*p* < 0.01, ∗∗∗*p* < 0.001. These data are representative of two independent experiments.

Epithelium mesenchymal transition (EMT) is a process associated with regulations in cell-cell adhesion proteins and loss of cellular polarity, where mesenchymal morphology is linked to the increase in migration and metastasis of cancer cells (21). Since the TGF-β–SMAD pathway is associated with cancer metastasis in models such as MDA-MB-231 breast cancer cells and LKB1 can prevent the activation of TGF-β–SMAD signaling, we looked at EMT markers (22). In SL + cell lines, the expression of epithelium cadherin (E-cadherin) was higher than the WT cell line (c2SL + vs cWT∗∗, c3SL + vs cWT∗∗) (Fig. 2, *A* and *B*; ∗∗*p* < 0.01). On the other hand, mesenchymal cadherin (N-cadherin) expression was higher in the WT clone (c2SL + vs cWT∗∗, c3SL + vs cWT∗∗; Fig. 2, *A* and *B*; ∗∗*p* < 0.01). The migration capacity was measured by scratch assay and a lower rate of migration was observed in edited cells after 24 h (c2SL + vs cWT∗∗, c3SL + vs cWT∗∗; Fig. 2, *C* and *D*; ∗∗*p* < 0.01). The clonogenic and survival potential of cells was determined by colony formation assay and the edited cells presented a reduction in both parameters (Fig. 2, *E*–*H*). The quantifications performed here were colony number (c2SL + vs cWT∗∗∗, c3SL + vs cWT∗∗∗; Fig. 2*F*; ∗∗∗*p* < 0.001); colony area (c2SL + vs cWT∗∗, c3SL + vs cWT∗∗; Fig. 2*G*; ∗∗*p* < 0.01); and absorbance of Violet Crystal incorporated in colonies (c2SL + vs cWT∗∗∗, c3SL + vs cWT∗∗∗; Fig. 2*H*; ∗∗∗*p* < 0.001).

### Super LKB1 enhances oxidative metabolism in A549-edited cells

**Figure 3:**
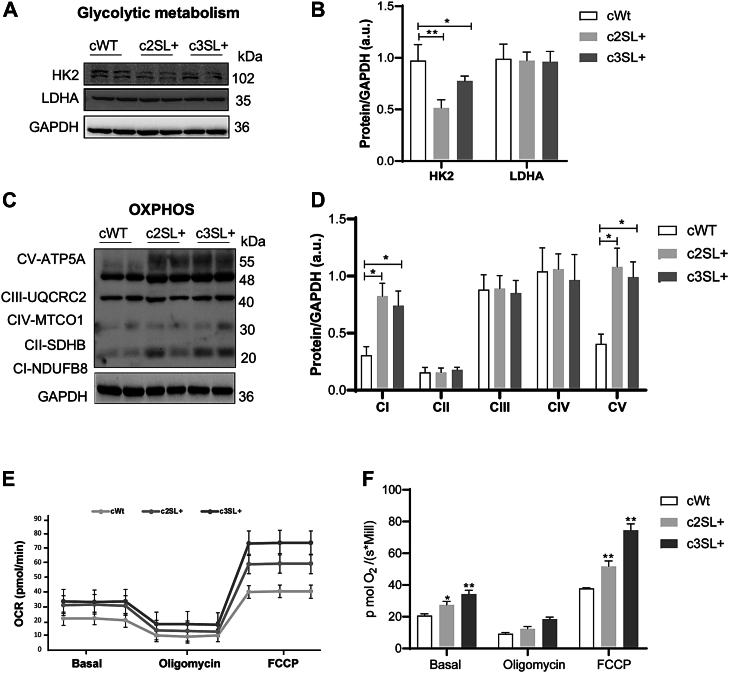
**Super LKB1 enhances the oxidative metabolism in A549-edited cells.***A*, Western blotting of HK2, LDHA, and GAPDH in cWT, c2SL+, and c3SL + cells; (*B*) normalized levels of proteins by GAPDH (a.u); (*C*) Western blotting of OXPHOS and GAPDH in cWT, c2SL+, and c3SL + cells. *D*, normalized levels of proteins by GAPDH (a.u); (*E*) oxygen consumption rate (OCR) by OROBOROS in cWT, c2SL+, and c3SL + cells; (*F*) comparison levels of O_2_ in the cell lines in the basal condition and after treatments with oligomycin and FCCP. The data are presented as mean ± SD. Statistical analysis was performed by ANOVA followed by Dunnet’s test ∗*p* < 0.05, ∗∗*p* < 0.01. These data are representative of two independent experiments.

Since AMPK is a central protein that regulates cellular metabolism (23), we investigated several metabolic parameters of A549-edited cells. The hexokinase 2 (HK2) and lactate dehydrogenase A (LDHA) proteins, associated with glycolytic metabolism, were analyzed and the HK2 expression was decreased in SL + A549 cells compared to WT cells (cWT vs c2SL+∗, cWT vs c3SL+∗∗) (Fig. 3, *A* and *B*; ∗*p* < 0.05, ∗∗*p* < 0.01). We also measured the expression of the mitochondrial oxidative phosphorylation system (OXPHOS), which is associated with oxidative metabolism. The A549-edited cells presented a higher expression of the OXPHOS, as highlighted by complex 1, which refers to NADH dehydrogenase [ubiquinone] 1 subcomplex, beta subunit 8, (c2SL + vs cWT∗, c3SL + vs cWT∗) (Fig. 3, *C* and *D*; ∗*p* < 0,05), and complex 5, referent to ATP-synthase 5A (c2SL + vs cWT∗, c3SL + vs cWT∗) (Fig. 3, *C* and *D*; ∗*p* < 0,05). Therefore, in response to the metabolism change, we performed Oroboros analysis as a functional experiment, evaluating the ratio of oxygen consumption of the cells. An increase in O_2_ consumption of SL + cells in comparison with WT cells in basal condition was observed (c2SL + vs cWT∗, c3SL + vs cWT∗∗; Fig. 3, *E* and *F*, ∗*p* < 0.05; ∗∗*p* < 0.01) and the challenge for maximum respiration (FCCP) (c2SL + vs cWT∗∗, c3SL + vs cWT∗∗∗; Fig. 3, *E* and *F*; ∗∗*p* < 0.01, ∗∗∗*p* < 0.001).

### The expression of Super LKB1 in A549-edited cells led to the inhibition of mTORC1 and an increase in autophagy signaling

**Figure 4:**
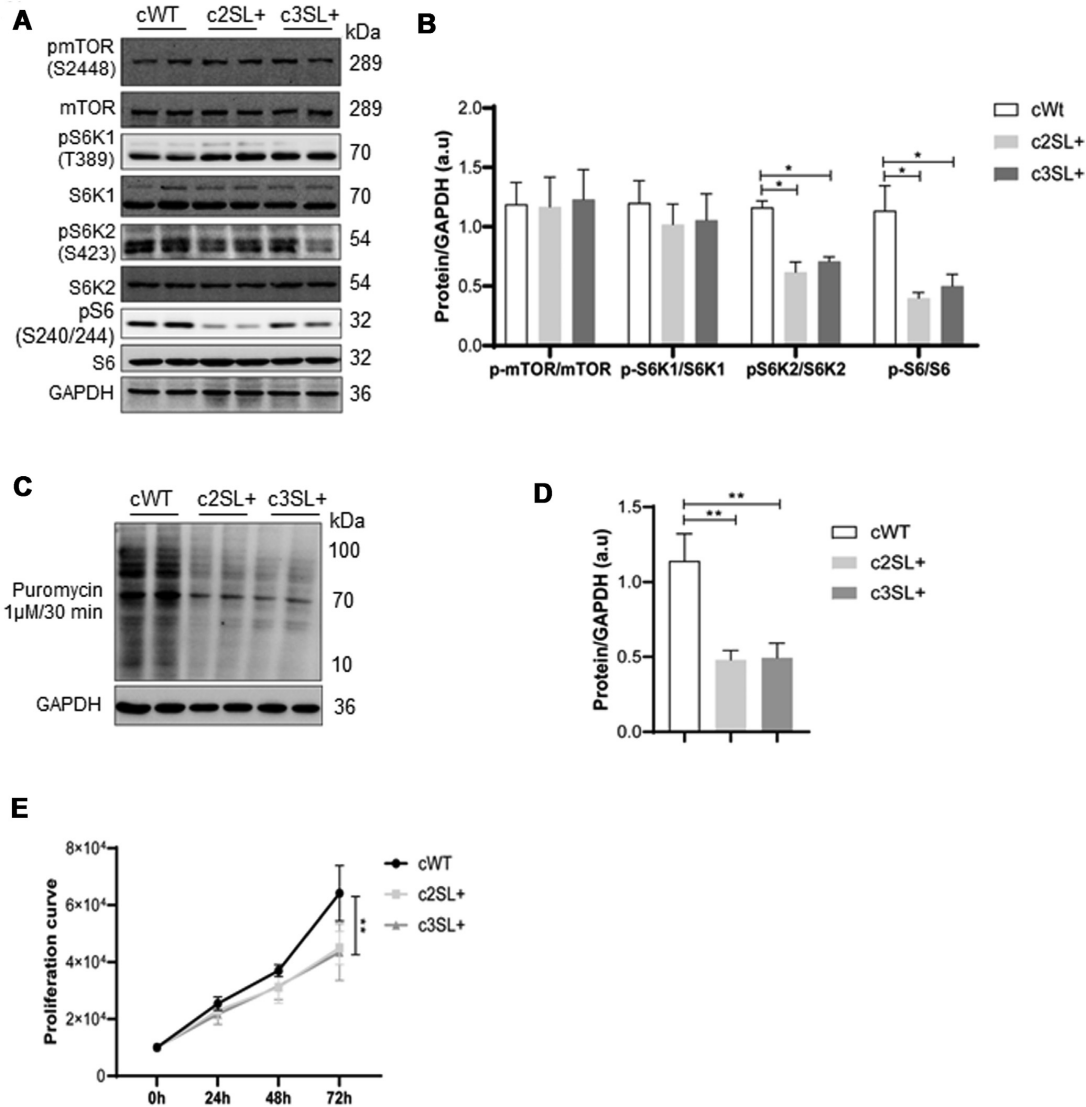
**The Super LKB1 inhibits mTORC1 effectors kinases, S6K2 and S6, impacting translation and proliferation activity.***A*, Western blotting of pmTOR (S2448), mTOR, pS6K1 (T389), S6K1, pS6K2 (S423), S6K2, pS6 (S240/244), S6, and GAPDH in cWT, c2SL+, and c3SL + cells; (*B*) normalized levels of proteins by GAPDH (a.u); (*C*) surface sensing of translation (SUnSET) by Puromycin treatment followed by Western blotting to detect puromycin-labeled amino acids in cWT, c2SL+, and c3SL + cells; (*D*) normalized levels of protein by GAPDH (a.u); (*E*) proliferation curve beginning with 1.10^4^ cells and counting for 72 h in cWT, c2SL+, and c3SL + cells. Data are presented as mean ± SD. Statistical analysis has been performed by ANOVA followed by Dunnet’s test ∗*p* < 0.05, ∗∗*p* < 0.01. These data are representative of two independent experiments.

Since AMPK can simultaneously activate the tuberous sclerosis proteins 1/2, which forms a protein complex that inhibits the Ras homolog enriched in brain GTPase, an activator of the mTORC1 (24), and the Unc-51–like autophagy-activating kinases 1 (ULK1) protein, which is a known activator of autophagy (25), we evaluated the mTORC1 and autophagy pathways in cells expressing the Super LKB1 protein. For mTORC1, edited cells presented a reduction in the activation of mTORC1 downstream targets: phospho-S6K2 serine 423 (cWT vs c2SL+∗, cWT vs c3SL+∗; Fig. 4, *A* and *B*; ∗*p* > 0.05) and phospho-S6 serine 240/244 (cWT vs c2SL+∗∗, cWT vs c3SL+∗; Fig. 4, *A* and *B*; ∗*p* < 0,05, ∗∗*p* < 0,01). Since the activation of mTORC1 is important for translation control (26), we performed the surface sensing of translation analysis to evaluate if the LKB1 expression could impact protein synthesis. WT cells incorporated more puromycin in 30 min compared to edited cells, which indicates that WT presents more protein synthesis than edited cells (cWT vs c2SL+∗∗, cWT vs c3SL+∗∗; Fig. 4, *C* and *D*; ∗∗*p* < 0.01). As mTOR and protein synthesis are directly involved with proliferation (27), a proliferation assay was performed over 72 h. The WT cells presented a higher number of cells than edited cells starting from an initial concentration of 1 × 10^4^ cells after 72 h (cWT vs c2SL+∗∗, cWT vs c3SL+∗∗; Fig. 4*E*; ∗∗*p* < 0.01).

**Figure 5:**
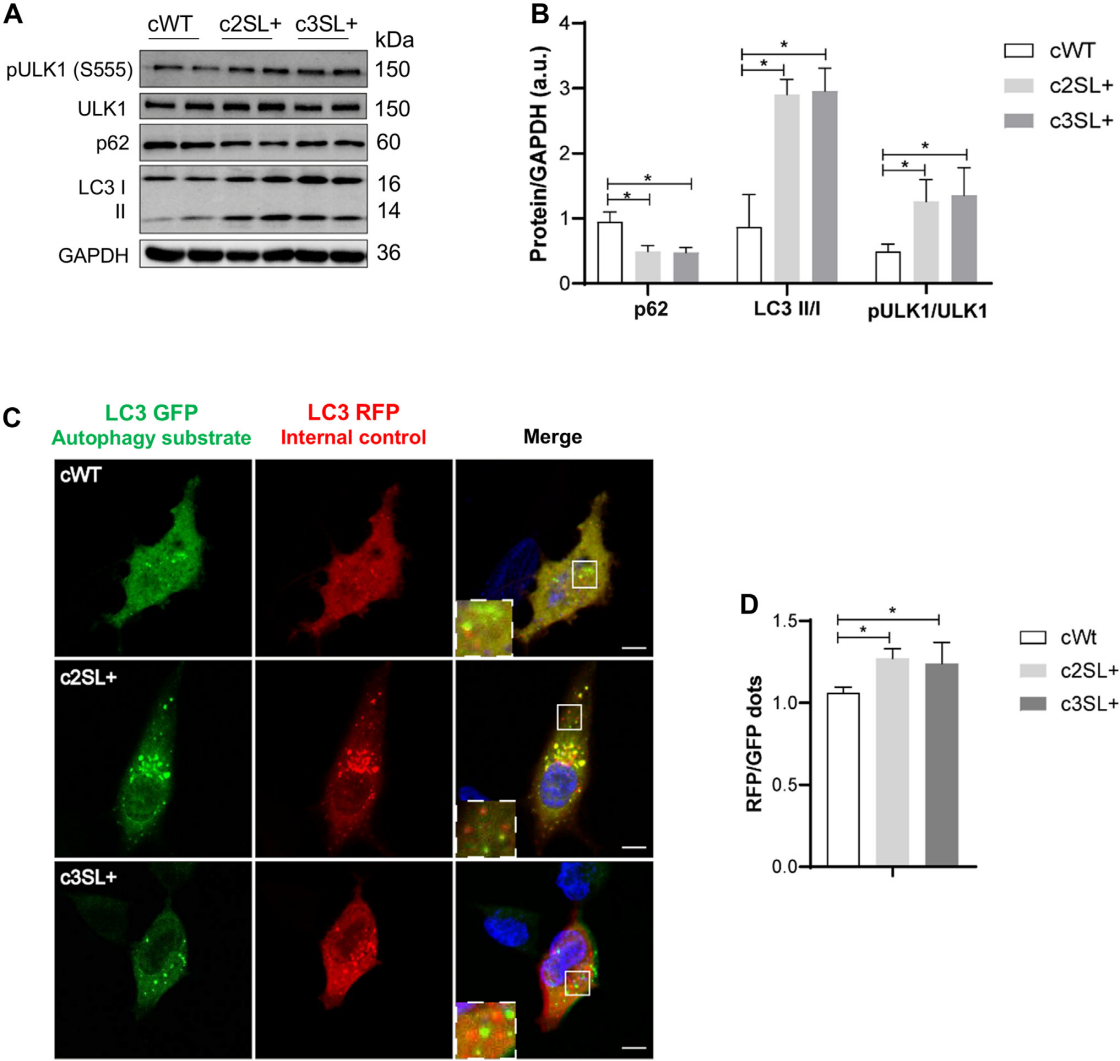
**The A549-edited cells show an increase in autophagy flux due to Super LKB1 expression.***A*, Western blotting of pULK (S555), ULK, p62, LC3 I/II, and GAPDH in cWT, c2SL+, and c3SL + cells. *B*, normalized levels of proteins by GAPDH (a.u). *C*, autophagy flux detection with ptfLC3 plasmid being LC3 GFP autophagy substrate and LC3 RFP internal control in cWT, c2SL+, and c3SL + cells, the highlighted square evidence the fluorescent dots; (*D*) normalized RFP/GFP in cWT, c2SL+, and c3SL + cells. The higher ratio indicates an increase in autophagy flux. The data are presented as mean ± SD. Statistical analysis was performed by ANOVA followed by Dunnet’s test ∗*p* < 0.05. These data are representative of two independent experiments.

Regarding the autophagy pathway, ULK1 activation was observed through the increase in serine 555 phosphorylation in LKB1 in edited cells (c2SL + vs cWT∗, c3SL + vs cWT∗; Fig. 5, *A* and *B*; ∗*p* < 0.05). Also, a reduction in p62 levels (c2SL + vs cWT∗, c3SL + vs cWT∗; Fig. 5, *A* and *B*; ∗*p* < 0.05) and an increase in microtubule-associated protein 1 A/1B-light chain 3 (LC3II) (c2SL + vs cWT∗, c3SL + vs cWT∗; Fig. 5, *A* and *B*; ∗*p* < 0.05) was observed in edited cells in comparison to WT cells. These findings suggest and are compatible with autophagy activation. To confirm, we used the ptflLC3 plasmid to evaluate if the edited cell presented an increase in autophagy flux. Super LKB1+ cells presented more red dots, exhibiting a higher RFP/GFP ratio and indicating more autophagy flux since LC3-RFP is internal control and LC3-GFP is an autophagy substrate (c2SL + vs cWT∗, c3SL + vs cWT∗; Fig. 5*C*; ∗*p* < 0.05).

### Autophagy impairs the NRF2 activation leading to a downregulation in the expression of antioxidant enzymes in the A549-edited cells

**Figure 6:**
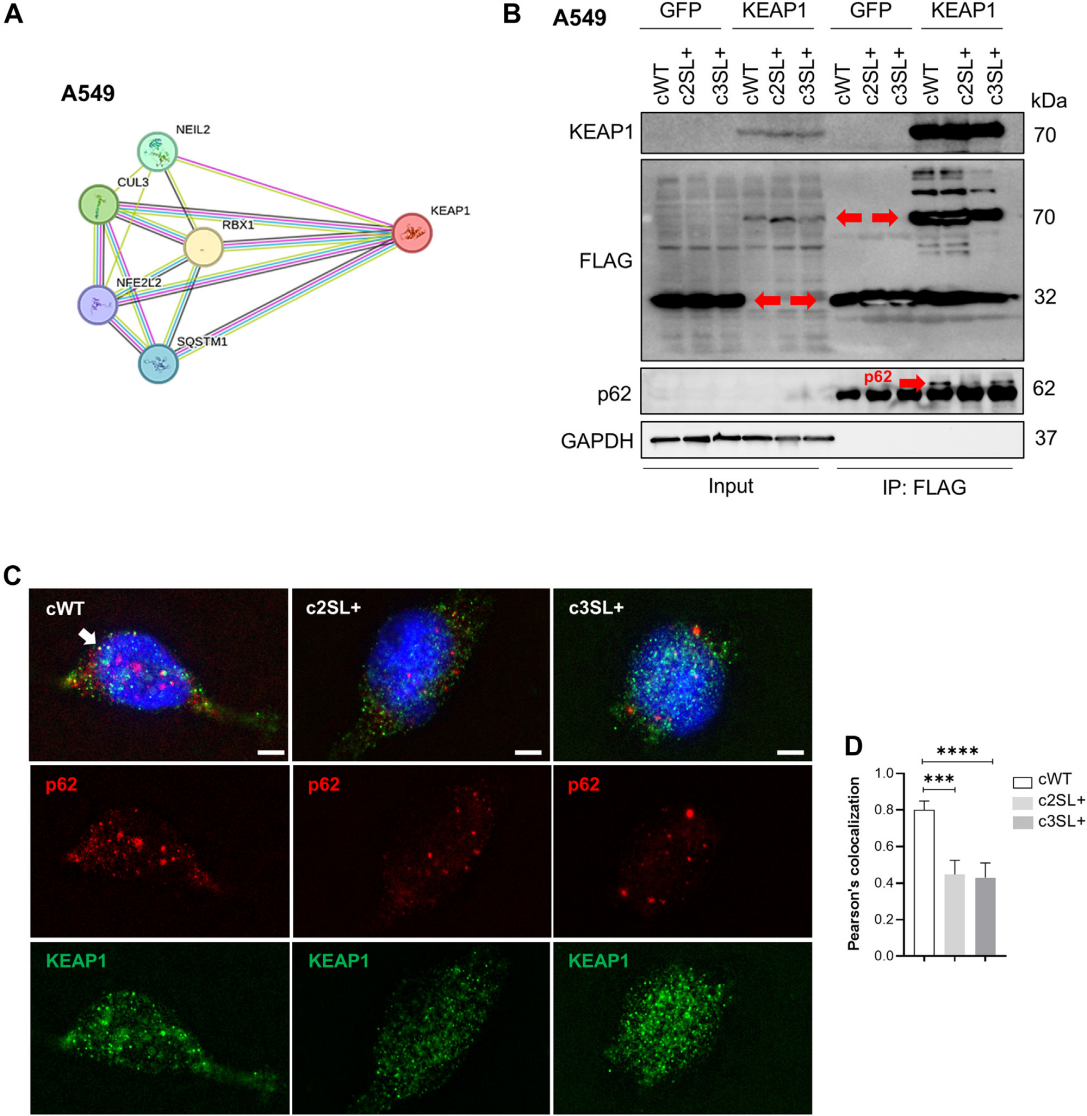
**The Super LKB1 expression increases the interaction between p62 and KEAP1 in A549 cells.***A*, protein–protein interaction (PPI) network by STRING software with highlight in the KEAP1 interaction. *B*, immunoprecipitation of A549-edited cells transfected with pFLAG-GFP or pFLAG-KEAP1, followed by Western blotting for p62, KEAP1, FLAG, and GAPDH. *Red arrows* indicate the target proteins FLAG-GFP and FLAG-KEAP1 and the interacting protein p62. *C*, immunofluorescence of p62 and KEAP1 in cWT, c2SL+, and c3SL + cells. *D*, quantification of colocalization cWT, c2SL+, and c3SL + cells by Pearson’s coefficient.

The activation of autophagy in the A549-edited cells seems to be relevant to redox balance, possibly due to the link between autophagy and redox pathways in the noncanonical activation of NRF2 (28). It is also known that KEAP1 can be sequestered in autophagosomes, which are prevented from interacting with lysosomes due to the inhibition of autophagy flux and the increased activation of mTORC1 (29). First, the interaction profile of the KEAP1 protein in human samples was analyzed using STRING, revealing an interaction between KEAP1, NRF2, and p62 (Fig. 6*A*). After that, pharmacological treatments were validated, metformin for autophagy stimulation through AMPK activation and 3-MA for autophagy inhibition. Metformin treatment decreased the p62 protein levels and increased the LC3II isoform, which indicates an increase in autophagy activity (Fig. S7*A*). With the combination of metformin and 3-MA in increasing concentration until 400 μM, an increase in the p62 protein levels and a decrease in LC3II expression were observed. (Fig. S7*A*). In HEK193T, immunoprecipitation targeting the interaction between p62 and KEAP1 was detected in the 3-MA treatment, corroborating the hypothesis that autophagy dysfunction can lead to KEAP1 and p62 interaction (Fig. S7*B*).

To assess whether a similar interaction profile between KEAP1 and p62 was observed in edited A549 cells, another FLAG immunoprecipitation was conducted. The results showed a stronger KEAP1–p62 interaction in the cWT cell line, which presented lower autophagic flux than the c2SL+ and c3SL + lines (Fig. 6*B*). To observe the colocalization of p62 and KEAP1, immunofluorescence was performed in A549-edited cells. The cWT cells exhibited a higher colocalization, as indicated by Pearson’s coefficient, compared to the c2SL+ and c3SL + cells, further supporting the interaction data between p62 and KEAP1 in cWT cells. (Fig. 6*C*).

We also evaluated the redox balance pathway in A549-edited cells, given the somatic mutation in the KEAP1 gene in A549 cells. We treated the cWT, c2SL+, and c3SL + cell lines with arsenite to assess HO-1 antioxidant enzyme expression and observed that cWT cells exhibited higher HO-1 expression than both A549-edited cells (cWT vs c2SL+∗∗, cWT vs c3SL+∗∗) (Fig. S7*C*; *p* < 0.001). Additionally, in the presence of the oxidant, all cell lines showed increased HO-1 expression (cWT vs cWT AsII∗, c2SL + vs c2SL + AsII∗∗, c3SL + vs c3SL + AsII∗∗) (Fig. S7*C*; ∗*p* < 0.05, and ∗∗*p* < 0.001), indicating that A549 respond to oxidative damage.

**Figure 7:**
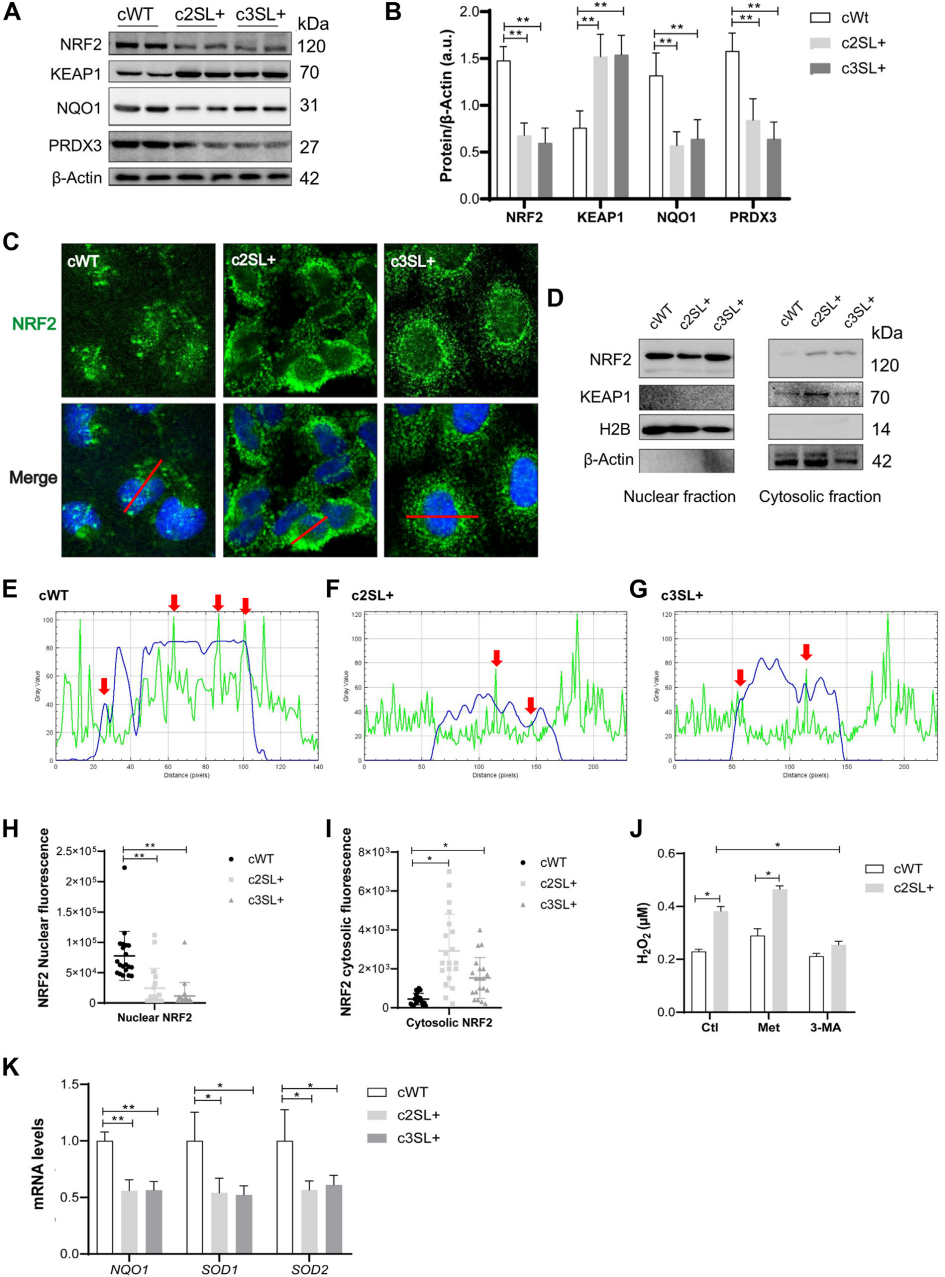
**The Super LKB1+ cells presented a cytoplasmatic localization of NRF2 and lower expression of antioxidant enzymes.***A*, Western blotting of NRF2, KEAP1, NQO1, PRDX3, and β-Actin in cWT, c2SL+, and c3SL + cells; (*B*) normalized levels of proteins by β-Actin (a.u); (*C*) immunofluorescence of NRF2 in cWT, c2SL+, and c3SL + cells; (*D*) subcellular fractionation of nucleus and cytosolic, followed by Western Blotting of NRF2, KEAP1, and β-Actin cWT, c2SL+, and c3SL + cells; (*E*) plot profile analysis of fluorescence distance in cWT cells, the *green* line refers to NRF2, and the *blue* line refers to the nucleus stained with DAPI. *F*, plot profile analysis of fluorescence distance in c2SL + cells, the *green* line refers to NRF2, and the *blue* line refers to the nucleus stained with DAPI. *G*, plot profile analysis of fluorescence distance in c2SL3+ cells, the *green* line refers to NRF2, and the *blue* line refers to the nucleus stained with DAPI. *Red arrows* in plot profiles represent peaks of signal colocalization. *H*, nuclear fluorescence quantification in cWT, c2SL+, and c3SL+. *I*, cytosolic fluorescence quantification in cWT, c2SL+, and c3SL+. *J*, relative H_2_O_2_ level (μM) in cWT and c2SL + cells in the control condition, and the treatments with metformin 10 mM and 3-MA 200 μM for 72 h. *K*, relative mRNA expression of NQO1, SOD1, and SOD2 in cWT, c2SL+, and c3SL + cells. The data are presented as mean ± SD. Statistical analysis was performed by ANOVA followed by Dunnet’s test ∗*p* < 0.05, ∗∗*p* < 0.01. These data are representative of two independent experiments.

Furthermore, a decrease in the expression of antioxidant enzymes NAD(P)H dehydrogenase [quinone] 1 (NQO1) and thioredoxin-dependent peroxide reductase (PRDX3) in Super LKB1 cells was observed, indicating impaired redox homeostasis (cWT vs c2SL+∗∗, cWT vs c3SL+∗∗; Fig. 7, *A* and *B*; ∗∗*p* < 0.01). The A549 WT cells presented a higher expression of NRF2 associated with lower KEAP1 levels, which is perhaps associated with the noncanonical activation of NRF2 in WT cells. On the other hand, edited cells presented a lower expression of NRF2 associated with a higher KEAP1 expression (cWT vs c2SL+∗∗, cWT vs c3SL+∗∗; Fig. 7, *A* and *B*; ∗∗*p* < 0.01). Immunofluorescence and subcellular fractionation were also performed to evaluate the localization of NRF2 (Fig. 7, *C* and *D*). Once in the nucleus, NRF2 is active and, in the cytoplasm, NRF2 is degraded by the proteasome (30). In the WT cells, nuclear signaling of NRF2 was detected (cWT vs c2SL+∗∗, cWT vs c3SL+∗∗; Fig. 7, *C* and *H*; ∗∗*p* < 0.01,) and, in Super LKB1+ cells, more cytoplasmatic signaling of NRF2 was found (cWT vs c2SL+∗, cWT vs c3SL+∗; Fig. 7, *C* and *I*; ∗*p* < 0.05).

To further quantify fluorescence, a plot profile analysis across the entire cell was performed. NRF2, marked by a green fluorescence signal, was detected in the nucleus due to its proximity to DAPI staining in all A549-edited cells as highlighted in the graphs (Fig. 7, *E*–*G*). However, in cWT cells, a notable enrichment of NRF2 near the DAPI signal was observed, suggesting stronger nuclear localization. These findings were corroborated by subcellular fractionation, which demonstrated higher NRF2 activation in cWT cells than c2SL+ and c3SL+ and greater cytoplasmic NRF2 expression in LKB1-edited cells (Fig. 7, *H* and *I*). These results were corroborated by subcellular fractionation, with a higher expression of NRF2 on cytoplasm fraction in LKB1-edited cells (Fig. 7, *D* and *I*).

To confirm that LKB1-edited cells presented impairment of redox control, we measured the mRNA levels of antioxidant enzymes: NQO1 (cWT vs c2SL+∗∗, cWT vs c3SL+∗∗; Fig. 7*K*; ∗∗*p* < 0.01), SOD1, and SOD2 (cWT vs c2SL+∗, cWT vs c3SL+∗; Fig. 7*K*; ∗*p* < 0.05). We, therefore, tested if the edited cells present reactive oxygen species (ROS) accumulation due to inhibition of the redox system. We observed that the edited cells presented higher concentrations of H_2_O_2_ (c2SL + vs cWT∗; Fig. 7*J*; ∗*p* < 0.05). When the cells were treated with metformin, an increase in H_2_O_2_ concentration in both cell lines was observed (cWT Ctl vs Met∗ and c2SL + Ctl vs Met∗; Fig. 7*J*; ∗*p* < 0.05). Finally, when we treated the cells with 3-MA, a decrease in H_2_O_2_ concentration was observed in the SL + cells (c2SL+ 3-MA vs Ctl∗; Fig. 7*J*; ∗*p* < 0.05). The H_2_O_2_ levels with 3-MA treatment were the same in WT A549 cells (Fig. 7*J*). Thus, the LKB1-edited cells seem to be more susceptible to oxidative damage, which can improve the response to cisplatin through H_2_O_2_ accumulation.

### The LKB1-edited cells accumulated more DNA damage and presented increased pro-apoptotic protein expression

**Figure 8:**
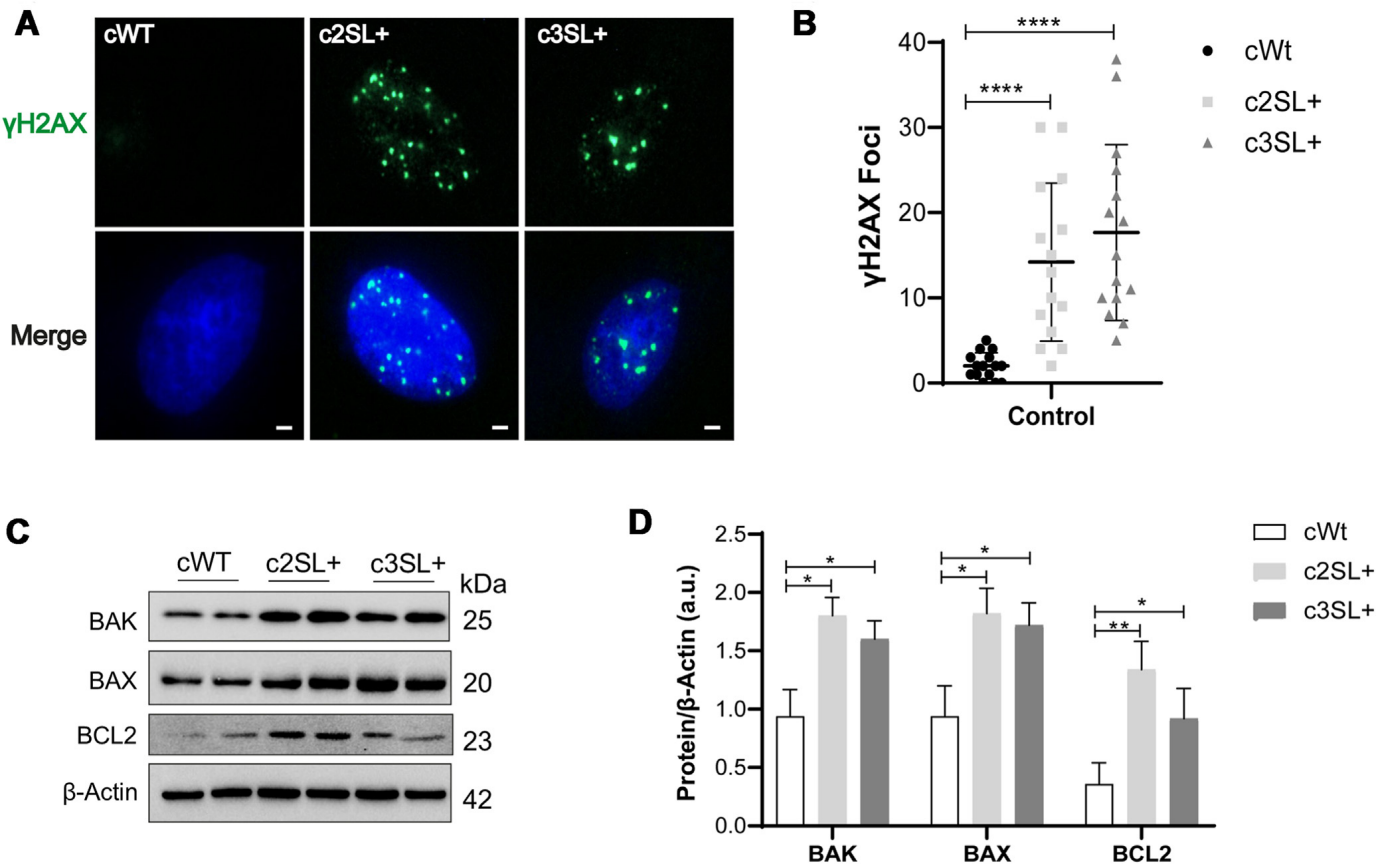
**Edited cells accumulated more DNA damage and higher pro-apoptotic protein expression.***A*, immunofluorescence of γH2AX in cWT, c2SL+, and c3SL + cells; (*B*) quantification of γH2AX foci in the nucleus of cWT, c2SL+, and c3SL + cells; (*C*) Western blotting of BAK, BAX, BCL2, and β-Actin in cWT, c2SL+, and c3SL + cells; (*D*) normalized protein level by β-Actin (a.u). The data are presented as mean ± SD. Statistical analysis was performed by ANOVA followed by Dunnet’s test ∗*p* < 0.05, ∗∗*p* < 0.01, ∗∗∗∗*p* < 0.0001. These data are representative of two independent experiments.

The oxidative damage in cells can recruit a DNA damage response for the maintenance of genomic homeostasis (31). Foci of γH2AX in the cell nucleus can indicate oxidative damage in the DNA of cells (32). The immunofluorescence of γH2AX in the LKB1-edited cells presented foci in the nucleus, but no foci were detected in the WT cells (c2SL + vs cWT∗∗∗∗, c3SL + vs cWT∗∗∗∗; Fig. 8, *A* and *B*; ∗∗∗∗*p* < 0.0001), suggesting more DNA damage in Super LKB1 than in WT cells. We also evaluated the apoptosis signaling due to oxidative damage in Super LKB1+ cells. An increased expression of Bcl-2 homologous antagonist/killer (BAK) and apoptosis regulator BAX in these cells (cWT∗, c3SL + vs cWT∗; Fig. 8, *C* and *D*; ∗*p* < 0.05) was detected. The antiapoptotic apoptosis regulator Bcl-2 (BCL2) was also increased in Super LKB1+ cells (c2SL + vs cWT∗∗, c3SL + vs cWT∗; Fig. 8, *C* and *D*; ∗*p* < 0.05, and ∗∗*p* < 0.001). Perhaps these findings could be related to autophagy since it induces antiapoptotic signaling (33).

### The A549-edited cells exhibited increased oxidative damage, rendering them more susceptible to cisplatin

**Figure 9:**
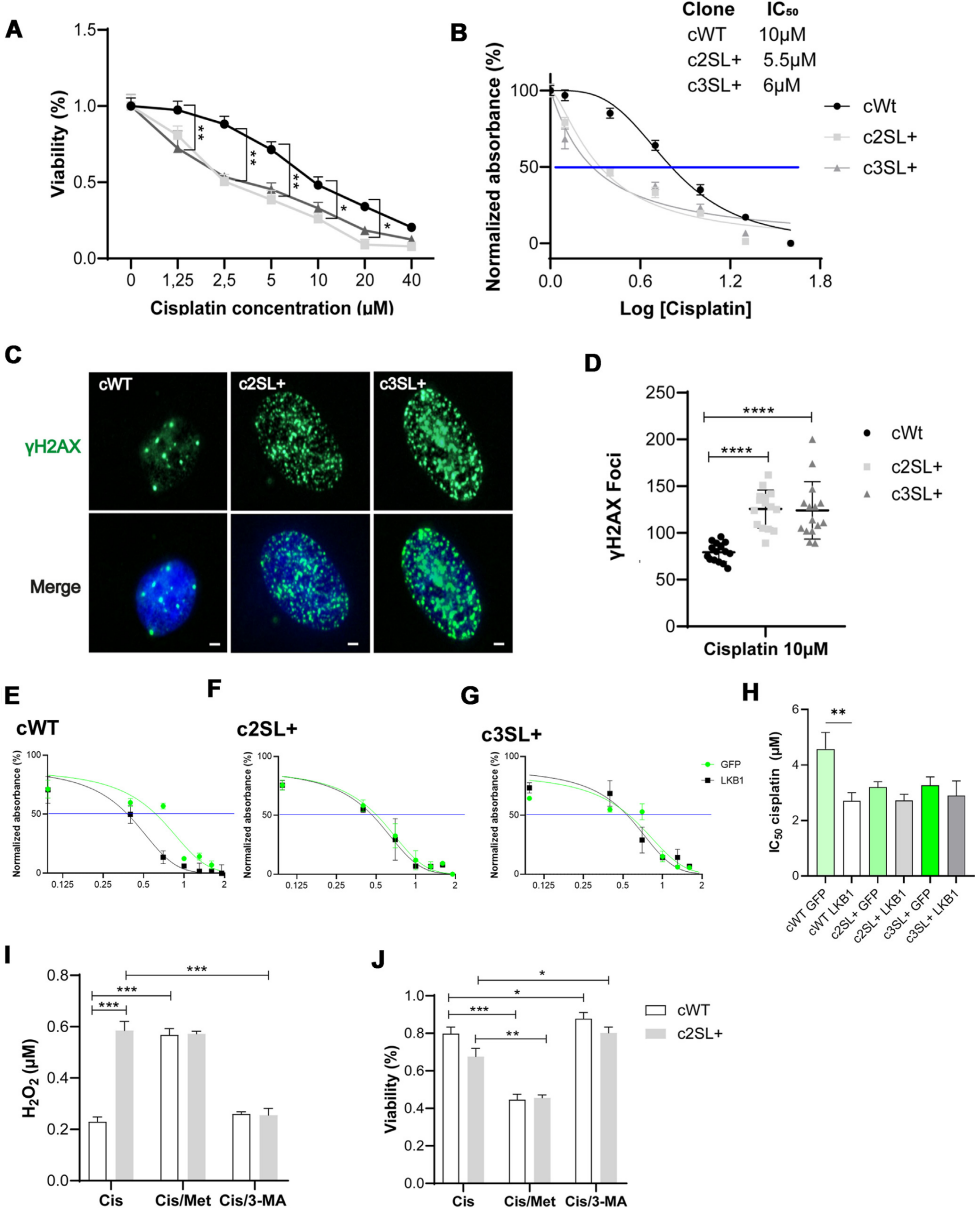
**ROS accumulated in edited cells improves cisplatin response.***A*, cWT, c2SL+, and c3SL + cells treated with several concentrations of cisplatin for 72 h followed by MTT to determine cell viability; (*B*) IC_50_ determination using PRISM Software; (*C*) immunofluorescence of γH2AX in cWT, c2SL+, and c3SL + cells treated with 10 μM of cisplatin; (*D*) quantification of γH2AX foci in the nucleus in cWT, c2SL+, and c3SL + cells; (*E*) IC_50_ determination in cWT cells comparing GFP versus LKB1 overexpression. *F*, IC_50_ determination in c2SL + cells comparing GFP versus LKB1 overexpression. *G*, IC_50_ determination in c3SL + cells comparing GFP versus LKB1 overexpression. *H*, quantification of IC_50_ in cWT, c2SL+, and c3SL+, comparing GFP versus LKB1 overexpression. *I*, H_2_O_2_ levels (μM) in cWT and c2SL + cells in the control condition, and the treatments with cisplatin, and combination with metformin 10 mM (Cis/Met) and 3-MA 200 μM (Cis/3 MA) for 72 h. *J*, MTT viability in cWT and c2SL + cells in the control condition, and the treatments with cisplatin, and combination with metformin 10 mM (Cis/Met) and 3-MA 200 μM (Cis/3-MA) for 72 h. The data are presented as mean ± SD. Statistical analysis was performed by ANOVA followed by Dunnet’s test ∗*p* < 0.05, ∗∗*p* < 0.01, ∗∗∗*p* < 0.001. These data are representative of two independent experiments.

Since cisplatin can induce ROS (34) and we demonstrated the oxidative DNA damage in Super LKB1 cells, the edited cells were challenged with cisplatin. First, the IC_50_ of cisplatin in the cell lines was determined, the WT cells presented a higher IC_50_: 10 μM compared to the edited cells, which presented IC_50_: c2SL+: 5.5 μM, c3SL+: 6 μM (Fig. 9, *A* and *B*). To evaluate the oxidative DNA damage after the cisplatin treatment, the γH2AX in the nucleus of cells was measured. In the LKB1-edited cells, more γH2AX foci were detected in comparison to WT cells (c2SL + vs cWT∗∗∗∗, c3SL + vs cWT∗∗∗∗; Fig. 9, *C* and *D*; ∗∗∗∗*p* < 0.0001). To evaluate whether the expression of canonical LKB1 could enhance the effects in A549-edited cells, LKB1 overexpression was performed, followed by determination of the cisplatin IC_50_ determination (Fig. 9, *E*–*G*). In cWT cells, the expression of canonical LKB1 reduced the cisplatin IC_50_, indicating sensitization (cWT GFP vs cWT LKB1∗∗). However, in the c2SL+ and c3SL + cells, the expression of canonical LKB1 did not alter the cisplatin IC_50_, showing no additional effects from the co-expression of canonical and Super LKB1 in A549-edited cells (Fig. 9*H*; ∗∗*p* < 0.001).

The c2SL + cells presented higher H_2_O_2_ amounts under cisplatin treatment in comparison to cWT cells (cWT Cis vs c2SL + Cis∗∗∗) (Fig. 9*I*; ∗∗∗*p* < 0.0001) and the combination of metformin with cisplatin increased H_2_O_2_ in both cells at the same level (Fig. 9*I*). On the other hand, the treatment with 3-MA and cisplatin decreased H_2_O_2_ in c2SL+ (cWT Cis/3-MA vs c2SL + Cis/3 MA∗∗∗) (Fig. 9*I*; ∗∗∗*p* < 0.0001). In the scenario of cellular viability, the combination of metformin with cisplatin decreased the viability in WT cells (cWT Cis/Met vs Cis∗∗∗; Fig. 9*J*; ∗∗∗*p* < 0.001). For c2SL + cells, the combination of metformin with cisplatin also decreased the viability (c2SL + Cis/Met vs Cis∗∗; Fig. 9*J*; ∗∗*p* < 0.001). Finally, when the cells were treated with the combination of 3-MA with cisplatin, a rescue of viability was observed in both cell lines (cWT Cis/3-MA vs Cis∗; Fig. 9*J*; ∗*p* < 0.05; c2SL + Cis/3 MA vs Cis∗∗; Fig. 9*J*; ∗∗*p* < 0.01). Thereby, autophagy activation seems to improve cisplatin response by H_2_O_2_ accumulation, and autophagy inhibition seems to inhibit cisplatin efficiency by H_2_O_2_ clearance, even in the presence of cisplatin treatment in A549 cells.

## Discussion

The mutated mRNA transcripts after the CRISPR/Cas9 edition can retain function, as observed in another work. In HAP1 cells with *TOP1* gene frameshift induced by CRISPR, the catalytic activity of the protein product was retained (17). The authors also generated the Super LKB1 in MIA cells, as a higher molecular weight isoform of the canonical LKB1. However, it is worth noting that functional data for Super LKB1 were not provided in their study. In the cell lines described here, we detected the presence of the alternative exon (131 nt), the +1 adenine insertion by CRISPR, and the (Q37∗, c.109C > T) in the A549 cells on the first LKB1 exon in the A549-edited cells (Fig. S3). Our study provides a proof of concept that NHEJ-based editing by CRISPR can be deployed to restore the loss of function of tumor suppressors.

This approach could be useful to induce genetic correction by the generation of a mutant protein with a similar function to the canonical WT isoform, which in the case of LKB1 could be useful to treat lung cancer. Multiple studies have demonstrated that the loss of LKB1 expression or function is directly associated with poor prognosis and chemotherapy resistance in lung cancer patients (35, 36). Additionally, a clinical study showed that LKB1 status impairs the efficacy of radiotherapy in stage III NSCLC. The data revealed lower overall survival in patients with reduced LKB1 expression, along with an inverse correlation between LKB1 expression and NRF2/NQO1 levels. This finding suggests that the worse prognosis in LKB1-deficient patients may be attributed to elevated antioxidant enzyme expression, driven by high NRF2 levels, which protect against radiation-induced DNA damage (37) Similarly, in our study, we observed a similar phenotype (Fig. 7*A*), where A549 WT cells exhibited higher NRF2/NQO1 expression than A549-edited cells.

Considering the poor outcomes for cancer patients associated with mutations and deregulation of tumor suppressor genes (38), the literature provides other examples of tumor suppressor gene restoration improving outcomes in experimental models. In leukemia, the CRISPR/Cas9 system was used to correct somatic mutations in the ASXL1 gene. This correction significantly impaired aberrant cellular signaling, reduced cell proliferation, and promoted myeloid differentiation. Additionally, it increased the survival of xenograft models injected with corrected cells compared to those injected with uncorrected cells (39). In colorectal cancer, transgenic mice were generated to enable conditional control of adenomatous polyposis coli protein expression *via* post-transcriptional regulation by shRNA. Tumor regression was observed following adenomatous polyposis coli restoration, accompanied by cellular differentiation and the re-establishment of crypt-villus homeostasis (40). The clinical relevance of tumor suppressor restoration has also been attested by successful examples, like the use of p53 in gene therapy approach using adenoviral vectors (41) and most recently has drawn attention again due to the development of mRNA delivery (42).

Other studies used the NHEJ-based CRISPR edition to generate functional proteins in other diseases besides cancer. One example is the targeting of the dystrophin gene. This gene is frequently afflicted with mutations in the 50-exon linked to duchenne muscular dystrophy, resulting in a nonsense sequence in subsequent exons and deletions in mRNA transcripts. The approach involved editing regions preceding the 50-exon mutation using CRISPR/Cas9. The strategy aimed to reframe the exon by introducing a +1 insertion in the DNA sequence, thereby generating a functional dystrophin protein (43). Additionally, exon skipping of the mutated exon was induced by targeting exon splicing enhancer sites through precise editing. This strategy improved the quality of skeletal muscle tissue in experimental models (44, 45), highlighting the potential of preserving the function and cellular signaling of mutated proteins as a CRISPR-based gene therapy approach.

As previously described, in the role of LKB1 as an activator of AMPK, numerous references emphasized its influence on the phosphorylation of the 172 threonine residue of AMPK by LKB1 and its interconnected pathways in metabolism and proliferation (46). In our work, we show that overexpression of canonical LKB1 increased the phosphorylation in the threonine 172 residue of AMPK in A549 WT cells (Fig. S5). The edited cells with expression of the Super LKB1 isoform also activated AMPK, like the canonical LKB1 (Fig. 1), thus increasing the activation of 172 threonine phosphorylation of AMPK (Fig. 1). AMPK activation has important tumor suppressor functions, inhibiting the mTOR pathway for example. mTOR was related to cisplatin resistance in A549 cells in a previous study of our group (47). It was demonstrated that metformin, a direct AMPK activator, induced lower levels of mTORC1, S6K1, and S6 protein phosphorylation in the combined treatment with cisplatin, besides improving the response to radiation in nonsmall lung cancer cells (48).

Several cancer characteristics, including the deregulation of cellular energetics, evasion of growth suppressors, and activation of invasion and metastasis were already described in different cancer types (49). These characteristics formed the basis for analyzing the suppressor activity of Super LKB1 in A549 cells. The HK2 enzyme expression plays a crucial role in tumor progression through the connection between the increased glucose uptake by cancer cells and the aerobic glycolysis, also known as the Warburg effect (50).

A study by Fang et al. demonstrated that inhibiting Glycogen synthase kinase-3 beta (GSK3β) in hepatocellular carcinoma (HCC) and Hep3B, liver cancer cells, led to a reduction in glucose uptake and HK2 expression. This inhibition increased AMPK activation through the phosphorylation of the 172 threonine residue, subsequently promoting the inhibition of mTORC1 and S6K1. Additionally, cells with GSK3β inhibition exhibited reduced clonogenic and migration activities, which underscore the tumor-suppressive potential of AMPK in cancer cells (51). In our work, the A549-edited cells that express the Super LKB1 exhibited lower clonogenic and migration potential (Fig. 2, *C* and *E*). There was also a reduction in HK2 (Fig. 3*A*) and mTORC1 expression (Fig. 4*A*) and an increase in oxygen consumption (Fig. 3*F*), which mirrors the previous findings regarding AMPK activation and suggests improvement from the Warburg effect shift of cancer cells.

AMPK is key in the interplay between mTORC1 and autophagy (52). Using HEK293T cells, we demonstrated that AMPK interacts with ULK1 and induces autophagy through serine 555 phosphorylation. However, mTORC1 can disrupt this interaction through the phosphorylation of ULK1 at serine 757. Another work also revealed that in HeLa cells overexpressing LKB1, AMPK-mediated ULK1 activation is crucial for autophagy flux and LC3II expression (53). Our findings here agree with the above study given the mTORC1 inhibition and increased phosphorylation of ULK1 at serine 555 by AMPK, promoting autophagy (Fig. 5).

It is established that A549 cells exhibit an addiction to NRF2, which is attributed to somatic mutations in the KEAP1 gene. These mutations result in a diminished interaction between KEAP1 and NRF2, leading to persistent and increased NRF2 activation (54). Furthermore, we demonstrated that arsenite treatment, which induces oxidative damage, can increase the expression of antioxidant enzymes such as HO-1 (Fig. S7*C*). Other studies have shown that modulation of KEAP1 expression through molecular tools, such as lentiviral knockdown, can elevate HO-1 expression, which is directly transcribed by NRF2 in A549 cells exposed to arsenic trioxide (55). Since NRF2 has an important role in the transcription activity of antioxidant enzymes, it was demonstrated that the NRF2 knockout in the DU-145 prostate cancer cell line can increase oxidative damage in cells, improving cisplatin effects (56).

LKB1/AMPK signaling is intricately connected to NRF2 and the regulation of oxidative stress, which has even been explored in clinical research (37). It is also worth mentioning that LKB1 and NRF2 have often mutated genes (or KEAP1 in the case of the NRF2 pathway) in lung cancer that may act in synergy to adapt cells to oxidative stress, as already demonstrated (57). It was also reported that, in HCC cells, the AMPK activator Acadesine/AICA riboside led to NRF2 activation and increased expression of antioxidant enzymes (58). While these findings underscore the association between LKB1/AMPK and NRF2, other pathways that influence the cellular redox balance have emerged, such as the impact of mTORC1-mediated p62 phosphorylation at serine 349 within the KIR domain (59). This mechanism of phosphorylation is prominent in cells with highly activated mTORC1 and low autophagy flux. It enhances p62 affinity for KEAP1, prompting NRF2 translocation to the nucleus and upregulating the expression of antioxidant enzymes in a noncanonical NRF2 activation pathway (18).

Our study validated the interaction between p62 and KEAP1 in HEK293T cells treated with the autophagy inhibitor 3-MA (Fig. S7). Besides, we show increased interaction between p62 and KEAP1 in the A549 WT cells compared to A549-edited cells (Fig. 6*B*). Additionally, in A549 WT cells characterized by elevated mTORC1 levels and low autophagy flux, we observed higher NRF2 levels (Fig. 7*A*) and increased antioxidant enzyme expression. In contrast, cells expressing Super LKB1, with lower mTORC1 and heightened autophagy flux, displayed lower NRF2 levels (Fig. 7*A*) and reduced antioxidant enzyme expression.

Cisplatin was more effective in Super LKB1+ cells (Fig. 9), which suggests that the lack of LKB1 and the high activity of NRF2 in A549 WT can promote resistance to oxidative damage by cisplatin. We hypothesize that the downregulation of NQO1, PRDX3, SOD1, and SOD2 ultimately leads to the accumulation of ROS levels. This hypothesis aligns with another work (60) that treated T24, bladder cancer cells, with cisplatin, detecting ROS through DCF staining and observing an intensification of apoptosis. Our results indicate that, under cisplatin treatment, cells with Super LKB1 expression exhibit an increased concentration of H_2_O_2_ compared to WT cells (Fig. 9), which supports the notion that the downregulation of antioxidant enzymes in A549 cells may contribute to elevated ROS levels, potentially influencing cellular responses to cisplatin-induced oxidative stress. A study using an expression vector for LKB1, delivered *via* liposomes in *in vivo* and *in vitro* models of NSCLC derived from A549 cells, demonstrated increased sensitivity to cisplatin and a reduction in cell proliferation (61), Similarly, these effects were observed with the expression of super LKB1 in A549 cells in our study.

**Figure 10:**
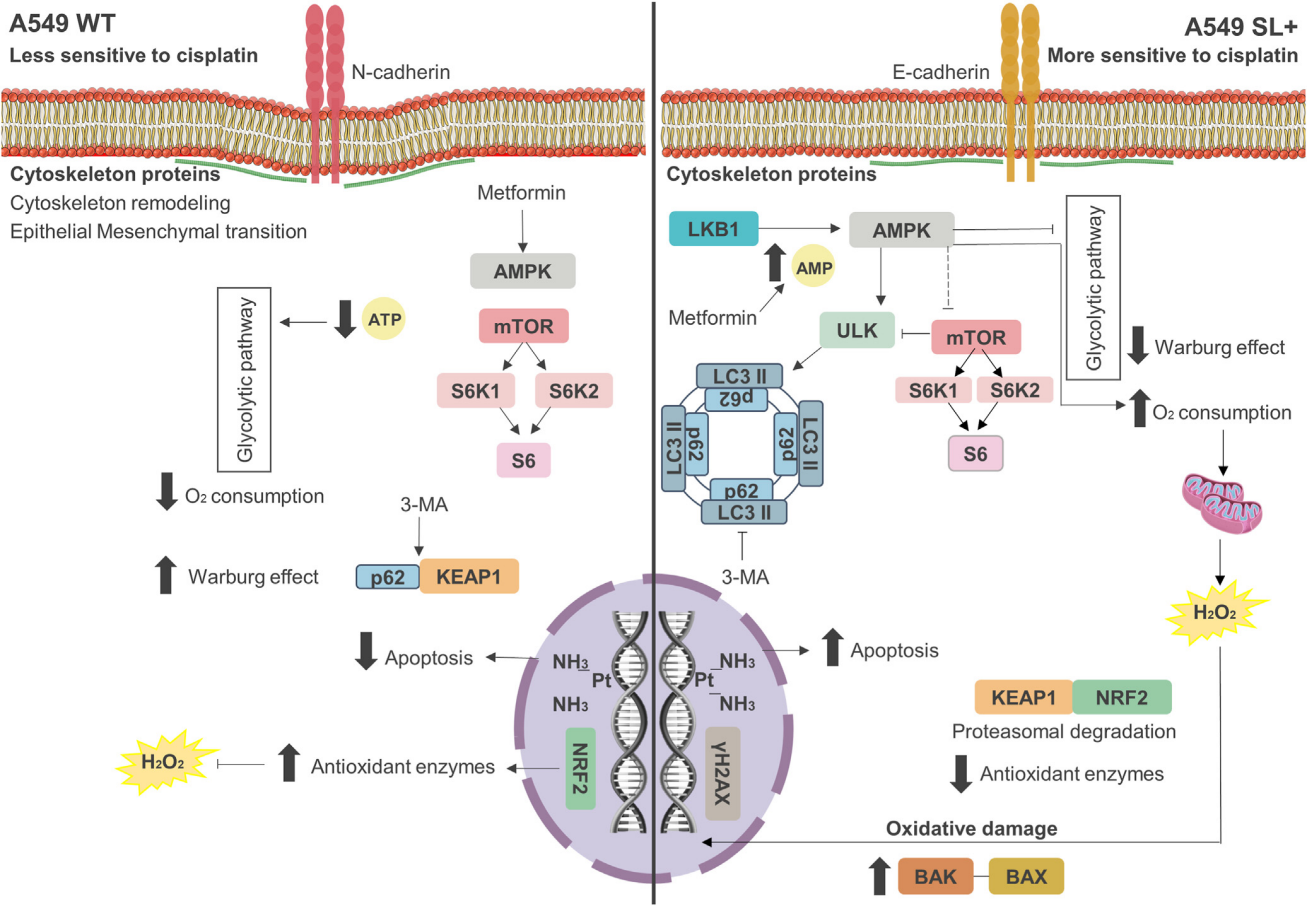
**The hypothesis of the potential role of Super LKB1 signaling in A549 in cisplatin treatment.** A possible mechanism of LKB1/AMPK signaling in the A549 cells based on data presented in the manuscript, comparing the cells that present A549 WT (LKB1 KO) and the A549-edited cells (Super LKB1) described here. In A549 WT, the cells have a lower AMPK activation, which leads to increase in mTORC1 signaling and protein synthesis and less autophagy flux. In this scenario, p62 can interact with KEAP1, promoting noncanonical activation of NRF2. The enhanced aerobic glycolytic metabolism may also contribute to the generation of NADPH by the pentose phosphate pathway and improve the expression of antioxidant enzymes, and the lack of LKB1 can contribute to the EMT process, increased migration, and survival. Thus, in A549 WT cells, activating redox pathways (such as NRF2) contributes to the diminished apoptosis signaling under cisplatin treatment. The Super LKB1 cells had increased activation of AMPK and increased autophagy flux. On the other hand, Super LKB1 cells presented decrease in mTORC1 pathway and protein synthesis. In this scenario, the interaction between p62 and KEAP1 is compromised, leading to NRF2 proteasomal degradation. Increased O_2_ consumption and oxidative metabolism can also contribute to oxidative damage, which may promote basal DNA damage, leading to cisplatin-induced apoptosis in edited cells. Finally, the Super LKB1 expression contributed to epithelium phenotype, reducing migration and survival in the edited cells.

In summary, we described the potential role of Super LKB1 signaling in A549 cells related to metabolism mTORC1 activity, autophagy, and redox homeostasis. The schematic in Figure 10 outlines a possible mechanism responsible for the response to cisplatin treatment in A549 NSCLC cells with LKB1 expression restoration. This study outlines the potential application of the *STK11* gene edition by NHEJ-CRISPR to restore LKB1 expression and consequently the cellular signaling of the AMPK pathway. Considering that *STK11* mutation occurs in around 17% of all cases of lung cancer, such an approach could potentially improve patients' quality for the type of cancer with the highest mortality rates worldwide.

## Experimental procedures

### Cell culture

A549 (human lung adenocarcinoma cell line) was cultivated in Ham’s F12K Medium (Sigma-Aldrich #N3520) supplemented with 10% fetal bovine serum (Gibco #12657029) and 1% penicillin/streptomycin (Gibco #15140-122). HEK293T (human embryonic kidney cell line that expresses a mutant version of the SV40 large T antigen) was cultivated in Dulbecco’s Modified Eagle Medium (Sigma-Aldrich #D7777) and supplemented with 10% fetal bovine serum and 1% penicillin/streptomycin. The cells were maintained at 37 °C in a humidified atmosphere containing 5% carbon dioxide.

### *STK11* (LKB1) edition by CRISPR/Cas9

First, in Benching software, we imported the human *STK11* sequence and we added the Q37∗, c.109C > T, which generates a PTC that is present in A549, which was validated by Sanger sequencing. CRISPOR software was used to select a high-specific and high-efficient sequence for an sgRNA adjacent to a PAM sequence (NGG) necessary for Cas9 activity (the sgRNA sequence was CCACCGCATCGACTCCACCG). The sgRNA was transcribed *in vitro* by EnGen sgRNA Synthesis Kit, *Streptococcus pyogenes* Kit (NEB #E3322 V) following the manufacturer’s instructions. The A549 cells were plated into a 6-well plate and, when a confluence of 70% was reached, the cells were transfected with Lipofectamine CRISPRMAX (Invitrogen #CMAX00003) and EnGen Spy Cas9 NLS (NEB #M0646 T), following the manufacturer’s instruction. A group of cells transfected without sgRNA was used as transfection control. To analyze the editing efficiency of the system, we performed the T7 endonuclease (T7E1) assay in the cell populations. After 48 h, the cells were then isolated into 96-well plates by seeding cells at low density using serial dilutions. The resulting monoclonal cultures were screened by Western blotting for the LKB1 expression using an LKB1 antibody (Cell signaling #3050). To evaluate the INDELs generated by the CRISPR/Cas9 system, the targeted genomic region for LKB1 was amplified by PCR. The primer sequence is in Table 1, from genomic DNA and sequenced by Sanger sequencing. The A549 WT clone were named cWT and the Super LKB1 clones were named c2SL+ and c3SL+. The possibility of off-targets occurrence was also evaluated. Two regions with similar sequences compared to the sgRNA–LKB1 targeted based on CRISPOR prediction, one in chromosome 6 and another in chromosome 10 were amplified by PCR and sequenced by Sanger; the primer sequence is in Table 2.

**Table 1:** Primers used in PCR to amplify the genomic locus of edition by CRISPR/Cas9

Primer	Sequence (5′-3′)
LKB1_genomic_F	TAGAACAATCGTTTCTGTTGAAGAAGGG
LKB1_genomic_R	CAGGGCATTTTAACTGGAGTCCAAGAG

**Table 2:** Primers used in PCR to amplify the genomic regions for off-target occurrence

Primer	Sequence (5′-3′)
LKB1_mm4_intergenic_RP11_chr10_F	CCCTGGGTCATACCACCAAG
LKB1_mm4_intergenic_RP11_chr10_R	TCCATCACAAGCCTGGGTTC
LKB1_mm4_exon_PFDN6/RGL2_chr6_F	GAGTGCGAGGAAGGGTTGG
LKB1_mm4_exon_PFDN6/RGL2_chr6_R	CTGTACTCCATCCACGCCTC

### Western blotting

Protein extracts from A549 cells were obtained using a cell lysis buffer (50 mM Tris–Cl pH 7.5, 150 mM NaCl, 1 mM EDTA, 1% Triton X-100, protease, and phosphatase inhibitor cocktail), and samples containing 40 μg were separated by SDS-PAGE and transferred into nitrocellulose membranes (Bio-Rad Laboratories, Inc.). The membranes were blocked in a solution of TBS-T (50 mM Tris–Cl, pH 7.5; 150 mM NaCl; 0.1% Tween-20) containing 5% of nonfat dry milk for 1 h with constant agitation. The membranes were incubated with primary antibodies overnight at 4 °C, washed five times with TBS-T, and incubated for 1 h with secondary antibodies at room temperature, followed by washing five times with TBS-T. The membranes were incubated with Pierce ECL Western blotting Substrate (Thermo Fisher Scientific #32106) to visualize the protein band in ChemiDoc Imaging System (Bio-Rad Laboratories, Inc.), and densitometry was performed using ImageJ software v1.53. The specificity of each antibody used was tested by comparing with the molecular weights of the ladder. Phosphorylated proteins were normalized by the total levels of interest proteins. Primary antibodies: LKB1 (Cell signaling, #3050), pLKB1 (Cell signaling, #3482), AMPK (Cell signaling #5831), pAMPK (Cell signaling #50081), p62 (Cell signaling #50081), LC3B I/II (Cell signaling #2775), S6 (Cell signaling #2317), pS6 (Cell signaling #9234), S6K1 (Cell signaling #5707), pS6K1 (Cell signaling #9234), S6K2 (Cell signaling #14130), pS6K2 (Invitrogen #PA5-105036), mTOR (Cell signaling #2972), pmTOR (Cell signaling #2971), GAPDH (Cell signaling #2118), E-cadherin (Cell signaling #3195), N-cadherin (Cell signaling #13116), OXPHOS (ab110413), β-Actin (Cell signaling #4970), NRF2 (Cell signaling #12721), KEAP1 (Cell signaling #4678), NQO1 (Cell signaling #3187), PRDX3 (Abcam #ab73349), BAK (Cell signaling #12105), BAX (Cell signaling #5023), BCL2 (Cell signaling #15071), FLAG (Cell signaling #14793), lactate dehydrogenase A (Abcam #ab52488), HK2 (Abcam #ab209847), H2B (Cell signaling #12364), puromycin (Sigma-Aldrich 12D10). Secondary antibodies: HRP-conjugated goat anti-mouse IgG (Sigma-Aldrich AP308P, 1:2000), goat anti-rabbit IgG (Sigma-Aldrich AP307P, 1:5000), and goat (Sigma-Aldrich A5420, 1:5000).

### Immunofluorescence

The A549 cells were plated in the density of 1 × 10^4^ cells in a 24-well plate and after treatments fixed with 4% formaldehyde for 15 min at room temperature, permeabilized with methanol for 10 min at 4 °C, then treated with blocking solution with 3% of bovine serum albumin, 0.1% Tween 20 in PBS 1× for 30 min at room temperature. Primary antibodies were added: LKB1 (Cell signaling #3050), pLKB1 (Cell signaling #3482), NRF2 (Cell signaling #12721), p62 (Cell signaling #5114), KEAP1 (Cell signaling, #8047), and γH2AX (Cell signaling #9718) overnight at 4 °C, washed five times with PBS 1×, and incubated with the secondary antibody anti-rabbit Alexa Fluor 594 (Invitrogen #A11012). The cells were incubated with Hoechst diluted 1:10,000 in PBS 1 × for 10 min for nuclei staining. Coverslips were finally mounted using Prolong (Invitrogen #P36980).

### Autophagy flux detection

The A549 cells were plated in the density of 1 × 10^4^ cells in a 24-well plate and transfected with ptfLC3 (Addgene #21074) using Lipofectamine (Thermo Fisher Scientific #18324012) and PLUS reagent (Thermo Fisher Scientific #11514015), following the manufacturer’s instruction. After 48 h, the cells were fixed with 4% formaldehyde for 15 min at room temperature and washed two times with PBS 1×. The cells were incubated with Hoechst diluted 1:10,000 in PBS 1× for 10 min for nuclei staining. Coverslips were finally mounted using Prolong (Invitrogen #P36980). The autophagy flux was measured through the ratio of red dots (RFP^+^) to green dots (GFP^+^).

### Wound healing: Scratch assay

The A549 cells were seeded at a density of 1 × 10^5^ cells in a 24-well plate, until confluence. Cells were treated with 60 μM mitomycin-C (Sigma-Aldrich #10107409001) for 2 h. Cell monolayers were scratched by a p200 sterile pipette tip, washed with PBS 1×, and incubated with Ham’s F12K supply with 10% SFB and 1% P/S. The images were captured at 0 and 24 h. The scratch area was analyzed under a light microscope (Optika Italy) using Optika Proview software.

### Colony formation assay

The A549 cells were seeded into 60 mm dishes at a density of 5 × 10^2^ cells, incubated for 10 days at 37 °C, and stained with violet crystal solution (0.05% violet crystal w/v, 1% formaldehyde, 1% PBS, and 1% methanol) for 20 min at room temperature. The number and size of colony cells were manually quantified. Demonstrative images of the colonies were obtained under an optical microscope (Optika) using Optika Proview software. The crystal violet crystal was eluted with isopropyl alcohol and the absorbance was measured in a spectrophotometer at 560 nm.

### Subcellular fractionation

From a p100 plate, 8 × 10^6^ cells were collected, centrifuged at 500*g* for 5 min, then washed with PBS 1× and again centrifuged at 500*g* for 5 min. The cell pellet was resuspended in 100 μl of CE buffer (10 mM Hepes, 60 mM KCl, 1 mM EDTA, 0.075% de NP40, 1 mM DTT, and protease inhibitor cocktail at pH 7.6), and the sample was incubated on ice for 3 min and then the cells were centrifuged at 1300*g* for 45 min at 4 °C. The supernatant constitutes the cytoplasmic extract of the cells, and the pellet is the nuclear extract. The supernatant (cytoplasm) was collected, and the pellet was washed with 100 μl of CE buffer without NP40; the nuclear extract was centrifuged at 1000*g* for 5 min and the supernatant was discarded. This procedure was repeated four more times. The nuclear pellet was resuspended in 100 μl of NE buffer (20 mM Tris–HCl, 420 mM NaCl, 1.5 mM MgCl_2_, 0.2 mM EDTA, 25% glycerol, and protease inhibitor cocktail at pH 8) and the sample was homogenized in the vortex for 1 min.

### DNA damage assay: Detection of phosphorylated foci of H2AX

The cells were seeded similarly to the immunofluorescence assay. After 48 h, the cells were incubated with 10 μM of cisplatin (Sigma-Aldrich PHR1624) or vehicle for the control group for 4 h. The cells were washed with PBS 1× and incubated with Ham’s F12K supply with 10% SFB and 1% P/S for 16 h for H2AX foci formation. The immunofluorescence protocol was then performed, as described.

### MTT and IC_50_

The A549 cells were plated at a density of 8 × 10^3^ cells in 96-well plates. After 24 h, the cells were incubated with cisplatin at 0, 1.25, 2.5, 5, 10, 20, and 40 μM (Sigma-Aldrich #PHR1624) for 72 h. The viability was measured by adding 10 μl of 12 mM MTT (Thermo Fisher Scientific #M6494) to each well and incubating for 2 h at 37 °C. The formazan crystals were solubilized in 1N HCl and isopropanol solution in a proportion of 1:25 for 20 min at 37 °C. The optical density was measured in a spectrophotometer at 570 nm. The IC_50_ was determined by a nonlinear regression on GraphPad Prism 8.01 software (https://www.graphpad.com/).

### H_2_O_2_ detection assay

The A549 cells were plated in 96-well plates. For H_2_O_2_ detection, the AmplexTM Red Hydrogen Peroxide/Peroxidase kit was used (Invitrogen A22188) following the manufacturer’s instructions.

### OROBOROS mitochondrial respiration

The cells were plated into a p100 plate and, after 72 h, 2 × 10^6^ cells were collected, washed with PBS 1×, and centrifuged at 500*g* for 5 min. The pellet was resuspended on miR05 buffer (0.5 mM EGTA, 3 mM MgCl_2_, 60 mM lactobionic acid, 1 mg/ml bovine serum albumin free fatty acid, 20 mM taurine, 10 mM KH_2_PO_4_, 20 mM Hepes, 110 mM sucrose at pH 7.1). The oxygen consumption rate measurement was taken at basal conditions, 1 mM oligomycin, and 1 mM FCCP in OROBOROS equipment (NextGen O2k).

### PCR, real-time quantitative PCR, and LKB1 cloning

The A549 cells were plated in a 6-well plate with a density of 1 × 10^5^ cells. Total RNA was extracted from the A549 cells using TRIzol (Thermo Fisher Scientific #15596026). The High-Capacity cDNA Reverse transcription kit (Thermo Fisher Scientific #4368814) was used to synthesize the cDNA. iTaq Universal SYBR Green Super Mix (Bio-Rad #10000068167) was used following the manufacturer’s instructions to perform real-time quantitative PCR. The gene expression was analyzed by the formula: 2^-ΔΔCt^, described by Livak in 1997, using β-actin as a housekeeping gene. Samples, in triplicate, were arranged in a 96-well plate (Applied Biosystems #4306737) for amplification and were run in the Step One Plus Real-Time PCR System (Applied Biosystems). The primer sequences used for RT-qPCR are presented in Table 3.

**Table 3:** Primers used in the RTqPCR experiments and the primers used to amplify the canonical LKB1 CDS that were cloned to overexpression

Primer	Sequence (5′-3′)
β-Actin qPCR Human F	GCCGCCAGCTCACCAT
β-Actin qPCR Human R	CCACGATGGAGGGGAAGAC
NQO1	AGGACCCTTCCGGAGTAAGA
NQO1	TGGAGATGTGCCCAATGCTAT
SOD1	GTTTCCGTTGCAGTCCTCG
SOD1	GGTCCATTACTTTCCTTCTGCTC
SOD2	AAGGAACGGGGACACTTACAAA
SOD2	AGCAGTGGAATAAGGCCTGTTG
LKB1 _cDNA_F_*Eco*RI	AAAGAATTCAATGGAGGTGGTGGACCCGCA
LKB1_cDNA_R_*Xho*I	AACTCGAGTCACTGCTGCTTGCAGGCCG

Abbreviation: cDNA, complementary DNA.

Underlined are restriction enzyme sites.

The coding DNA sequence of canonical LKB1 was amplified with Platinum Taq DNA Polymerase (Thermo Fisher Scientific #10966018) following the manufacturer’s instructions. The PCR product was purified with the PureLink PCR Purification Kit (Thermo Fisher Scientific #K310001). The cDNA of HEK293T cells was used as a template for canonical LKB1 cloning and the primer sequences for LKB1 cloning are in Table 2. For PCR, the High-Fidelity DNA Polymerase (NEB #M0491S) was used following the manufacturer’s instructions. The PCR product was cloned into a pGEM T Easy vector (Promega A1360), digested with *Eco*RI and *Xho*I, purified with the PureLink PCR Purification Kit, and then ligated into a pcDNA-FLAG.

### Bioinformatic analysis

The Benchling web server (https://www.benchling.com/) was used to align the DNA sequence, the DECODR (https://decodr.org/) web server was used to decompose the Sanger sequencing, the Expasy web server (https://www.expasy.org/) was used to translate the DNA sequence into an amino acid sequence, and the Clustal Omega (https://www.ebi.ac.uk/jdispatcher/msa/clustalo) was used to align the amino acid sequences of the cWT and c2SL + cell lines.

The AlphaFold Protein Structure Database (https://colab.research.google.com/github/sokrypton/ColabFold/blob/main/AlphaFold2.ipynb) (62) was used to predict the protein structure of LKB1 and Super LKB1, the RCSB Protein Data Bank was used to import the canonical LKB1 sequence, and the PyMOL software (www.pymol.org/pymol) was used to visualize the proteins. The b-factor coloring was used to exclude low-confident regions in the structures.

The TargetP2.0 (https://services.healthtech.dtu.dk/services/TargetP-2.0/) and MULocDeep web servers (https://www.mu-loc.org/) were used to predict the localization of the isoforms. The STRING web server (https://string-db.org/) was used to generate the protein–protein interaction net of the LKB1 and the ClusPro protein–protein docking (https://cluspro.org/help.php) was used to demonstrate the interaction between AMPK and the canonical LKB1 or the Super LKB1, considering electrostatics and clustering to predict the most plausible interactions.

### Proliferation curve assay

The A549 cells were plated into a 6-well plate at a density of 1 × 10^4^ cells and manually counted with trypan blue exclusion after 24, 48, and 72 h.

### Anti-FLAG immunoprecipitation

Cells seeded in a p100 were transfected with pFLAG-GFP or pFLAG-KEAP1 with Lipofectamine following the manufacturer’s instruction. After 48 h, the A549 protein extracts were collected. For HEK293T, cells were incubated with 10 mM metformin (Sigma-Aldrich #D150959), 200 μM 3-MA (Sigma-Aldrich #M9281), or a vehicle for the control group for 24 h. Protein extract was collected, 40 μg was used as input, and the remaining protein extract was incubated with 50 μl of washed anti-FLAG resin (Millipore #A4596), per group, overnight. The beads were centrifuged at 8200*g* for 1 min and washed 5 times with TBS (50 mM Tris–Cl, pH 7.5; 150 mM NaCl). Finally, the proteins were denatured with Laemmli buffer with 10% of β-mercaptorethanol (Thermo Fisher Scientific #21985023), followed by a Western Blotting assay.

### Pharmacological treatments (cisplatin, metformin, 3-MA, puromycin, and arsenite)

For the viability assays, the anti-FLAG immunoprecipitation, and the H_2_O_2_ experiment, cells were treated with 10 μM cisplatin, 10 mM metformin, and 200 μM 3-MA for 72 h. For the surface sensing of translation assay, the A549 cells were treated with 1 μM puromycin for 30 min, for the assessment of oxidative stress in A549 cells, they were treated with arsenite 25 μM for 4 h and the protein was extracted from the cells, as described in the Western blotting section.

### Statistical analysis

Statistical analysis was performed using GraphPad Prism 8.01 software (https://www.graphpad.com/) and all data were expressed as mean and SD. The difference between means was tested by Student's *t* test, one-way or two-way ANOVA, followed by post hoc of Tukey and Dunnet, in which ∗*p* < 0.05, ∗∗*p* < 0.01, ∗∗∗*p* < 0.001, and ∗∗∗∗*p* < 0.0001 was considered significant.

## Conclusions

This study generated a functional higher molecular weight LKB1 isoform by CRISPR-Cas9. The A549 WT cell line presented increased redox activation in comparison to Super LKB1–edited cells, which may explain why the A549 WT cells are less sensitive to cisplatin. We explored a possible mechanism of LKB1/AMPK signaling that contributes to cisplatin and finds metabolic, mTORC1, and autophagy regulations. These pathways can be related to redox balance and NRF2 activation by the noncanonical activation of NRF2. Thus, the Super LKB1+ cells accumulate more oxidative damage, which may contribute to a better response to cisplatin treatment in comparison to WT cells. Importantly, the co-expression of canonical LKB1 does not enhance the response to cisplatin in the A549 cells with Super LKB1 expression, suggesting redundant protein activity. The pharmacological combination of metformin (an autophagy inducer) with cisplatin seems to contribute to the increase of H_2_O_2_ in WT cells. Combining 3-MA (an autophagy inhibitor) with cisplatin seems to rescue the viability of Super LKB1+ cells. This study characterizes the effects of a novel LKB1 isoform generated by CRISPR/Cas9 in A549 and its impact on metabolic targets and cellular pathways, showing the feasibility of using NHEJ repair mediated by CRISPR to rescue the expression of tumor suppressors. This research may contribute to future therapeutic strategies for enhancing cisplatin treatment in lung cancer that presents specific mutations in the LKB1 gene.

## Data availability

Complete datasets are available at the Zenodo repository (https://doi.org/10.5281/zenodo.11177239) upon request.

## Supporting information

This article contains supporting information (15).

## Conflict of interest

The authors declare that they have no conflicts of interest with the contents of this article.
