# Supplementary figures and images for: A CRISPR-edited isoform of the AMPK kinase LKB1 improves the response to cisplatin in A549 lung cancer cells
Source: J Biol Chem. 2025 Feb 13;301(3):108308. doi: 10.1016/j.jbc.2025.108308 (PMC11952844; doi:10.1016/j.jbc.2025.108308)

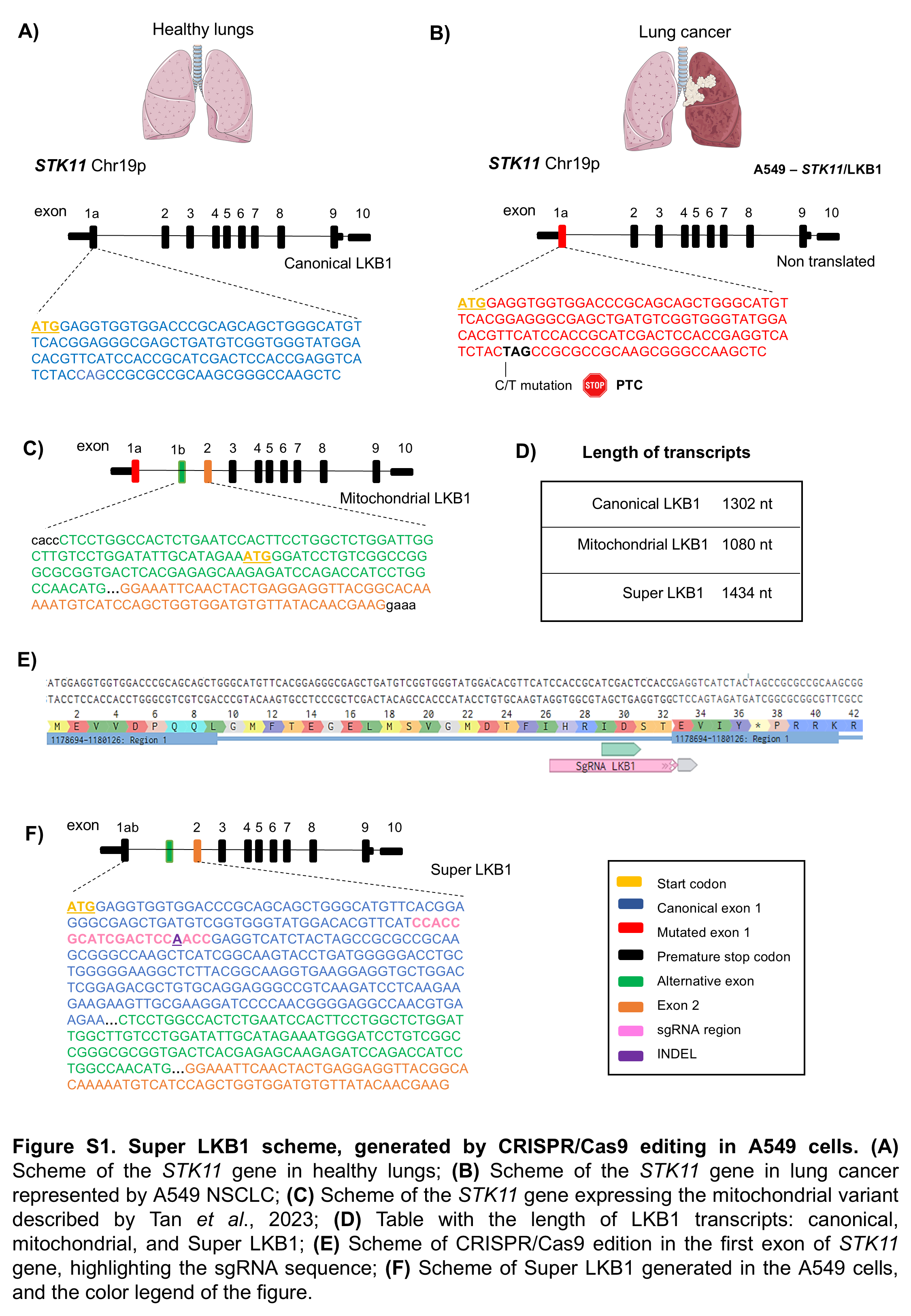

Supplement: Figure S1–S8 [file mmc1.zip › Support information/Figure S1.TIF]

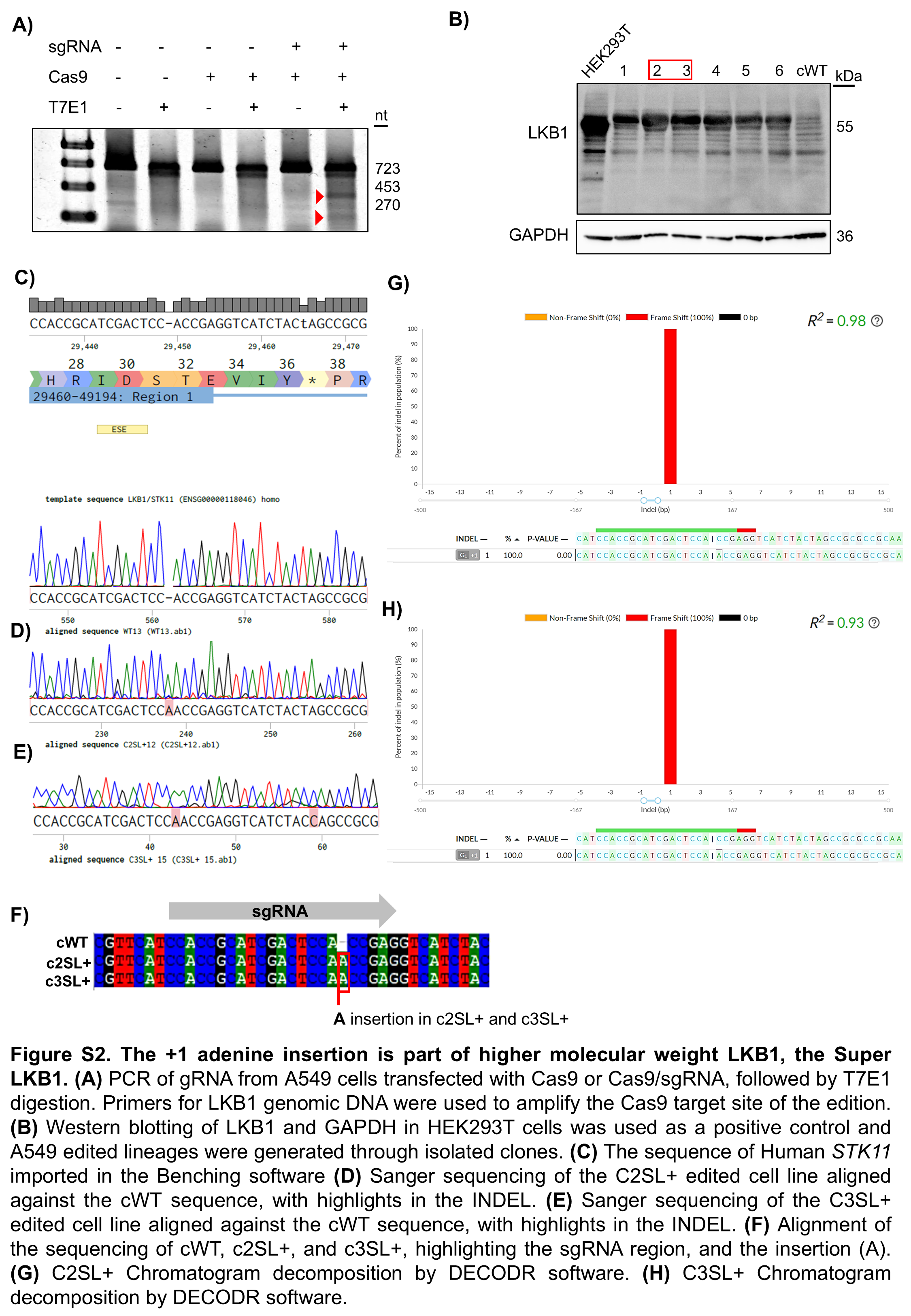

Supplement: Figure S1–S8 [file mmc1.zip › Support information/Figure S2.TIF]

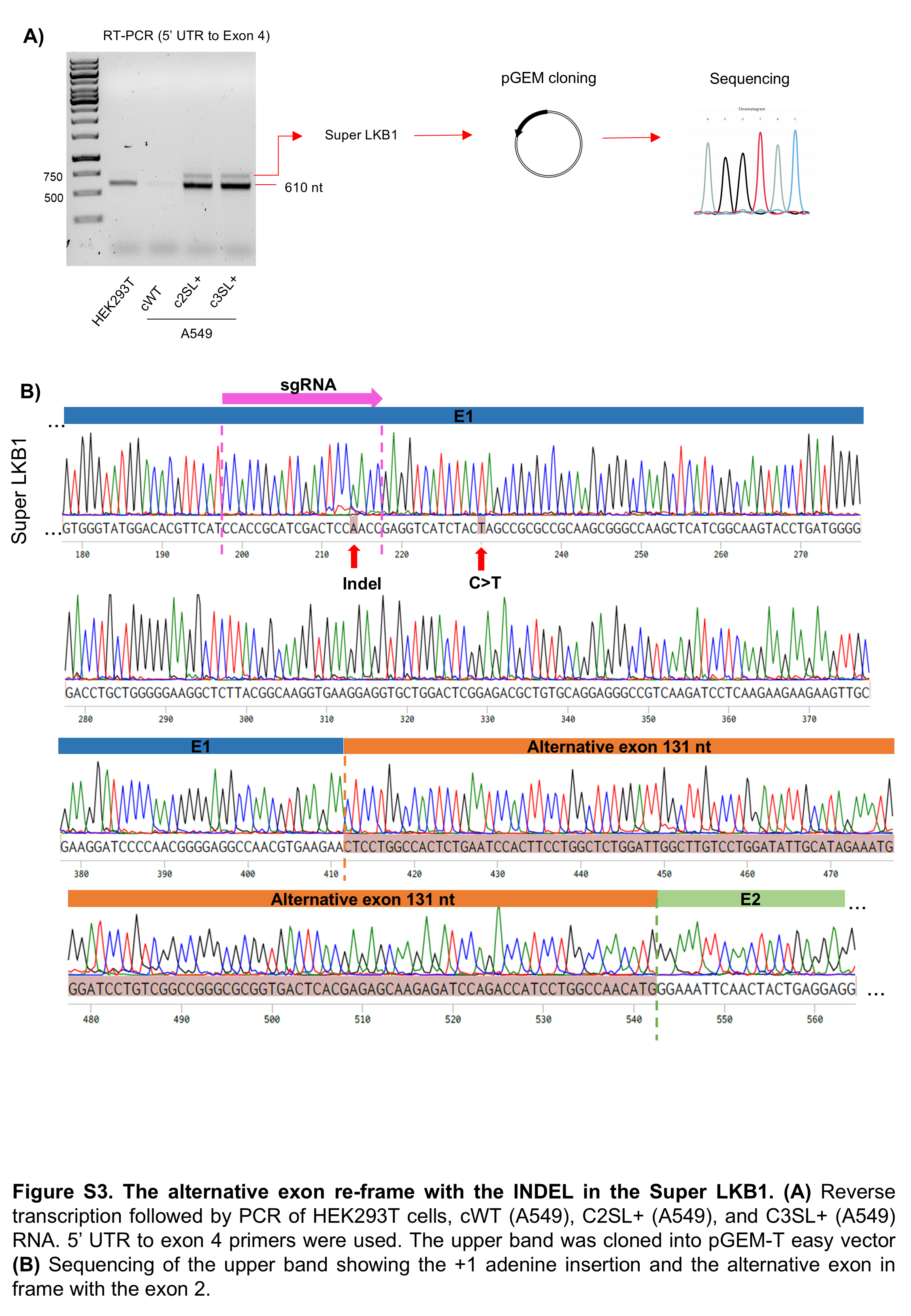

Supplement: Figure S1–S8 [file mmc1.zip › Support information/Figure S3.TIF]

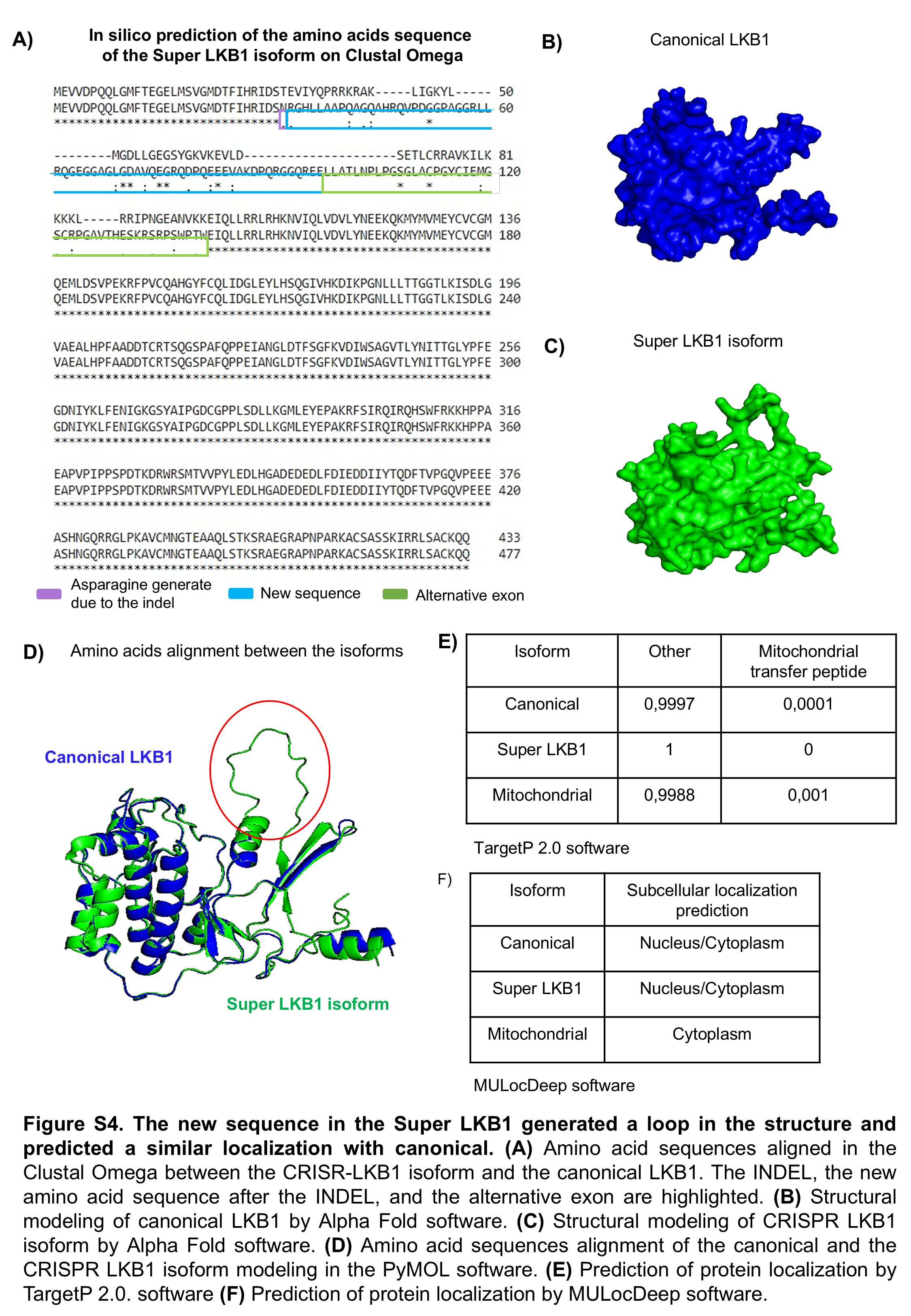

Supplement: Figure S1–S8 [file mmc1.zip › Support information/Figure S4.TIF]

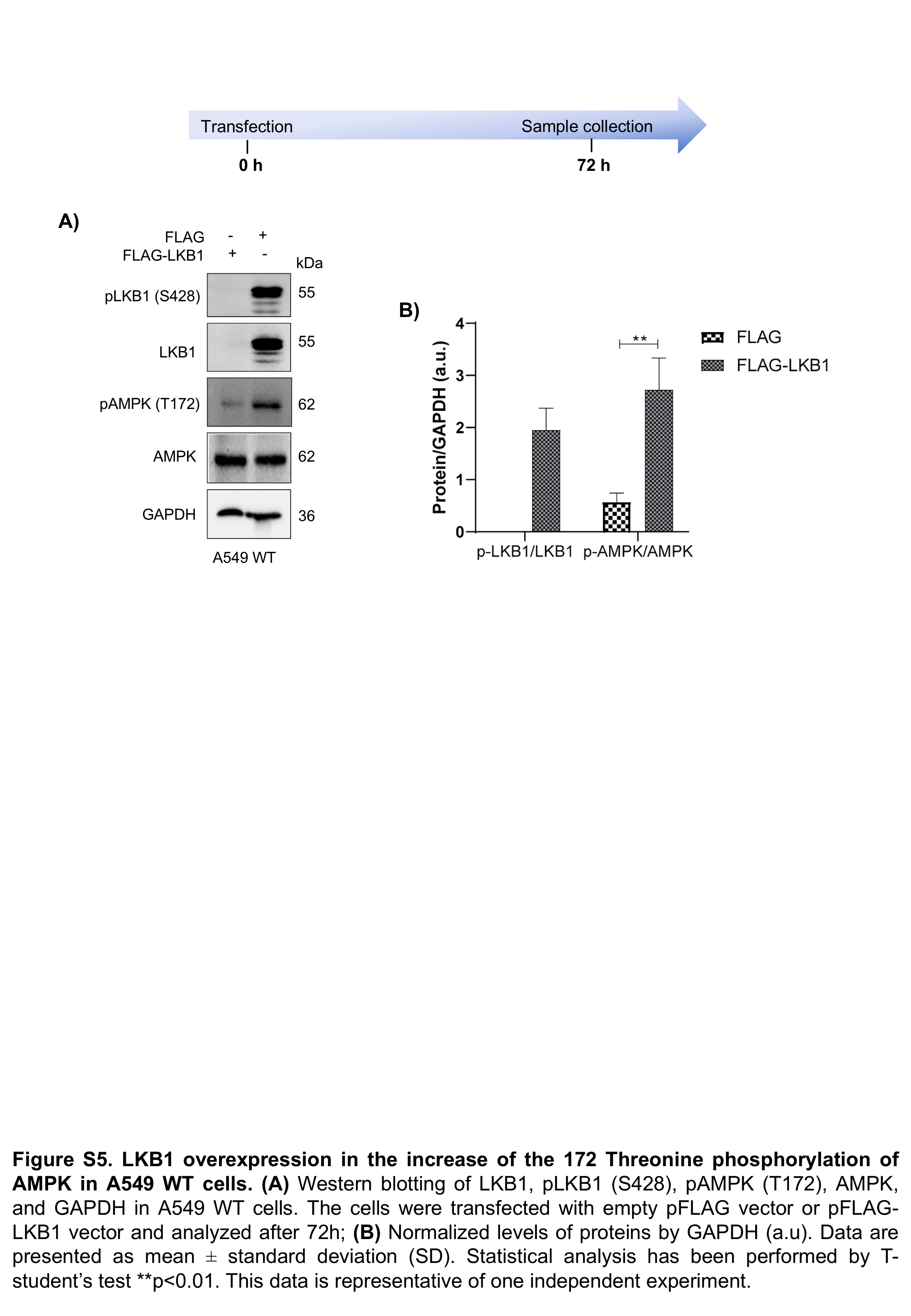

Supplement: Figure S1–S8 [file mmc1.zip › Support information/Figure S5.TIF]

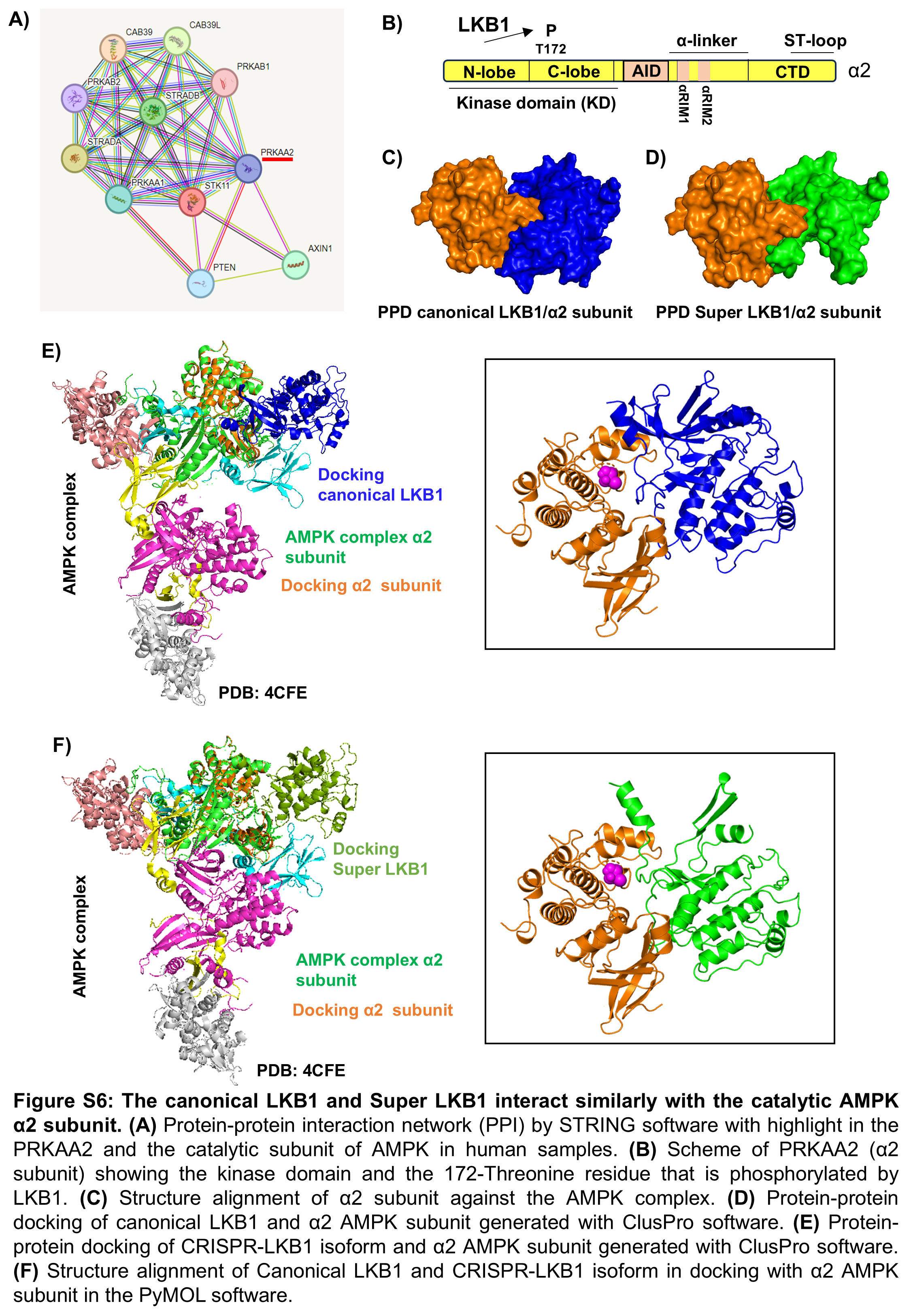

Supplement: Figure S1–S8 [file mmc1.zip › Support information/Figure S6.TIF]

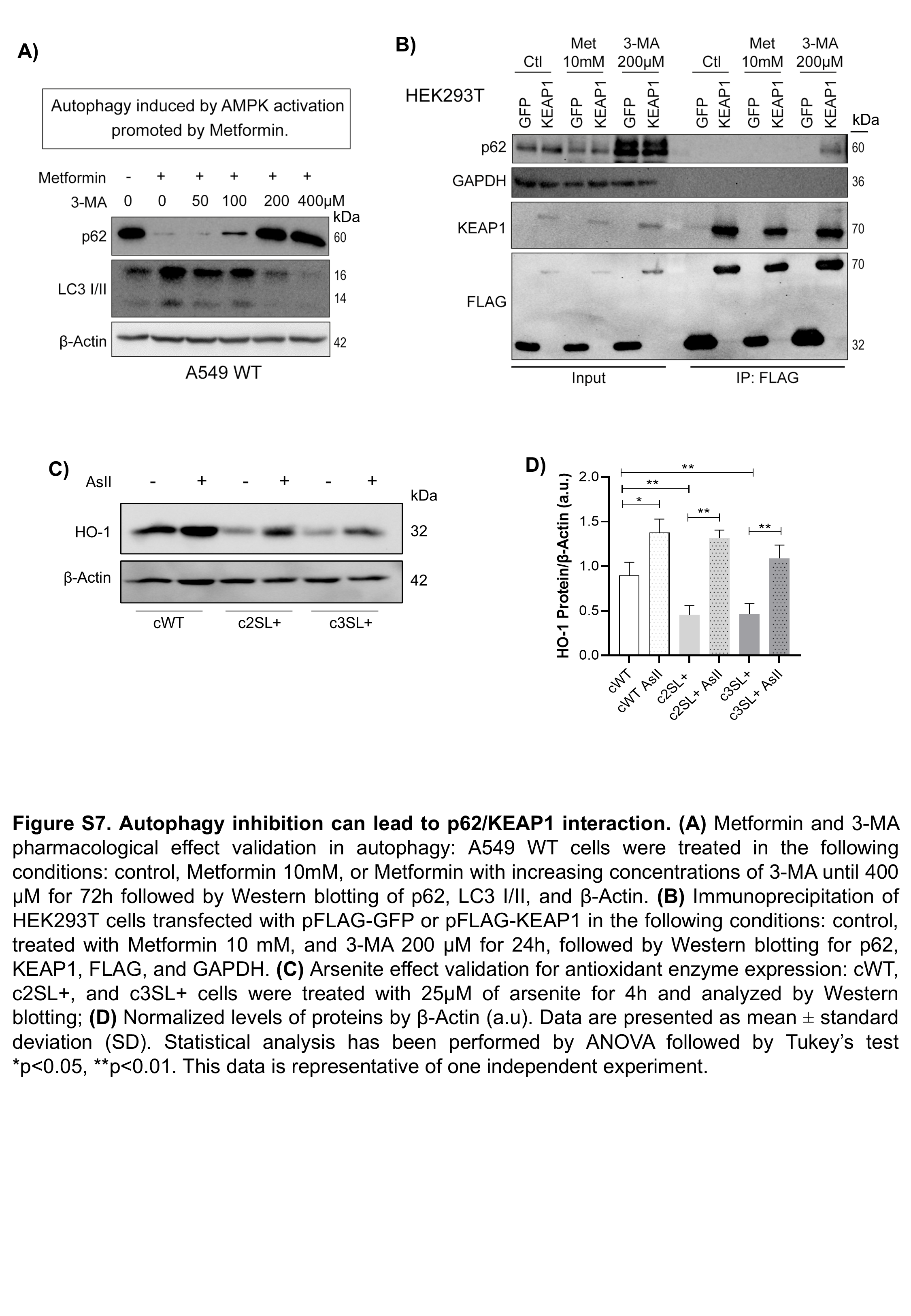

Supplement: Figure S1–S8 [file mmc1.zip › Support information/Figure S7.TIF]

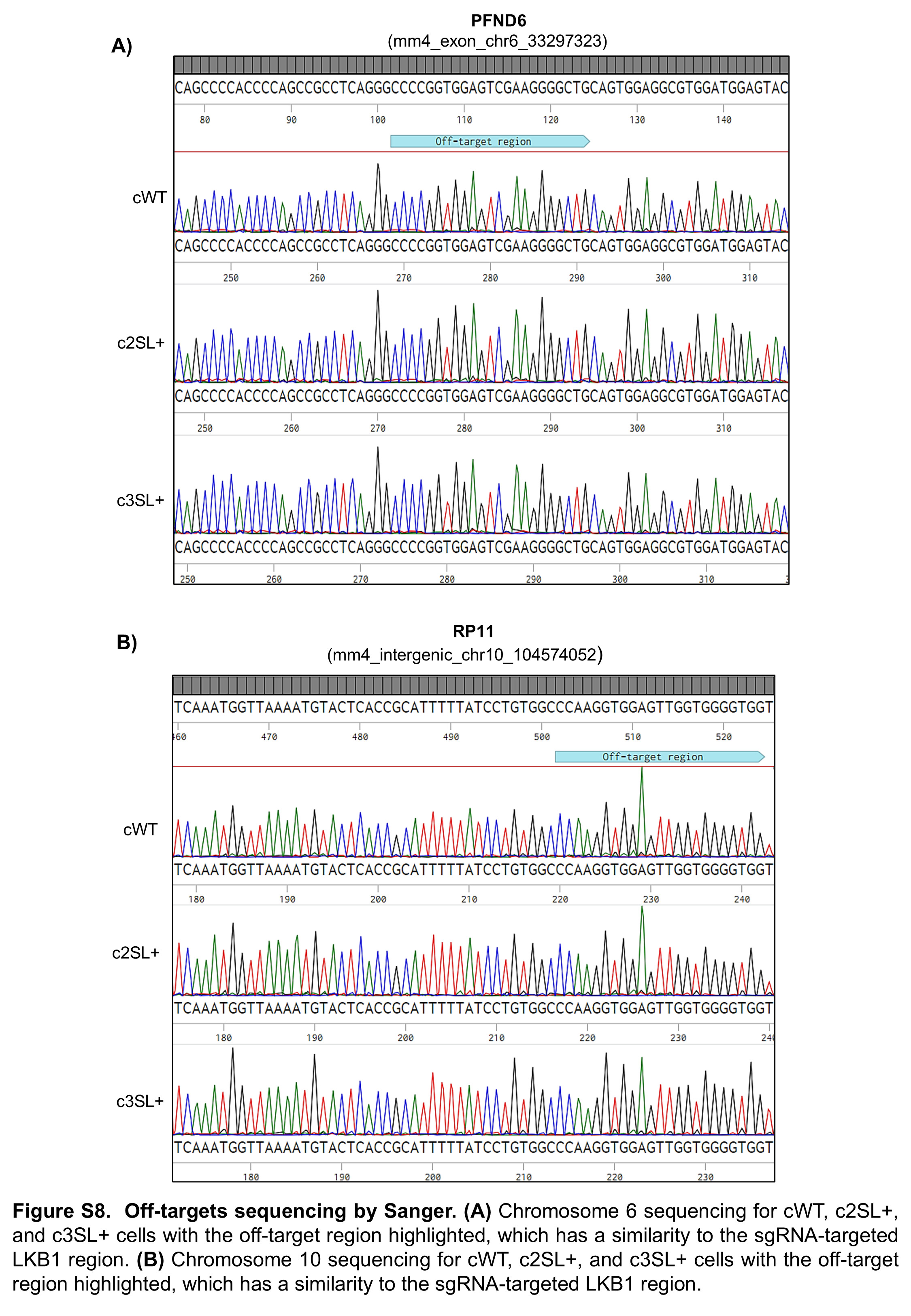

Supplement: Figure S1–S8 [file mmc1.zip › Support information/Figure S8.TIF]
